# Long-term continuous monitoring of methane emissions at an oil and gas facility using a multi-open-path laser dispersion spectrometer

**DOI:** 10.1038/s41598-023-50081-9

**Published:** 2024-01-05

**Authors:** Rutger IJzermans, Matthew Jones, Damien Weidmann, Bas van de Kerkhof, David Randell

**Affiliations:** 1 Shell Global Solutions International B.V., Grasweg 31, 1031 Amsterdam, The Netherlands; 2 Space Science and Technology Department, STFC Rutherford Appleton Laboratory, Didcot, OX11 0QX UK; 3 MIRICO Ltd., Unit 6, Zephir Building, Harwell Campus, Didcot, OX11 0RL UK

**Keywords:** Optical sensors, Climate-change mitigation, Natural gas, Power distribution, Statistics

## Abstract

A method for methane emissions monitoring at industrial facility level was developed based on a high precision multi-open-path laser dispersion spectrometer combined with Bayesian analysis algorithms using Monte Carlo Markov Chain (MCMC) inference. From the methane path-averaged concentrations spatially distributed over the facility under study, together with the wind vector, the analysis allows detection, localization and quantification of fugitive methane emissions. This paper describes the very first long term (3 months), continuous (24 h/7 days) deployment of this monitoring system at an operational gas processing and distribution facility. The continuous monitoring system, made of the combination of the open-path high-precision (<10 ppb) methane concentration analyser and the data analysis method, was evaluated with controlled releases of methane of about 5 kg/h for short periods of time (30–60 min). Quantification was successful, with actual emission rates lying well within the quoted uncertainty ranges. Source localisation was found to lack accuracy, with biases of 30–50 m in the direction of the line of sight of the spectrometer, due to the short duration of the controlled releases, the limited wind vector diversity, and complications from air flows around buildings not accounted for by the transport model. Using longer-term data from the deployment, the MCMC algorithm led to the identification of unexpected low intensity persistent sources (<1 kg/h) at the site. Localisation of persistent sources was mostly successful at equipment level (within ~20 m) as confirmed by a subsequent survey with an optical gas imaging (OGI) camera. Quantification of these individual sources was challenging owing to their low intensity, but a consistent estimate of the total methane emission from the facility could be derived using two different inference approaches. These results represent a stepping stone in the development of continuous monitoring systems for methane emissions, pivotal in driving greenhouse gas reduction from industrial facilities. The demonstrated continuous monitoring system gives promising performance in early detection of unexpected emissions and quantification of potentially time-varying emissions from an entire facility.

## Introduction

With a global warming potential of 84 (28) over 20 (100) years and a perturbation lifetime of 12 years in the atmosphere, methane (CH_4_) is a more potent greenhouse gas than carbon dioxide (CO_2_)^1^. CH_4_’s atmospheric lifetime is so short compared to CO_2_’s (hundreds of years) that reducing methane emissions offers immediate potential for mitigating global warming on a timescale compatible with the 2015 Paris Agreement goals. The global background level of methane in the atmosphere is about 1.92 ppm on average, increasing at more than 0.01 ppm/year, primarily due to human activity^2^. Recent (2017) bottom up models suggest that more than 60% of total methane emissions are anthropogenic; oil and gas production, distribution and transport accounts for approximately 12% of total methane emissions into the atmosphere^3^. With warming, positive climate feedback from natural sources is also expected to occur^4^.

Currently, the best practice in the oil and gas sector is to use targeted leak detection and repair campaigns to reduce fugitive emissions^5^ from facilities, with the detection and localisation process typically done through snapshot, labor-intensive surveys using optical gas imaging (OGI) cameras. Best practices for emissions reporting are given in the OGMP 2.0 reporting framework^6^: a voluntary, comprehensive, measurement-based reporting framework for the oil and gas industry, which recommends periodic source-level and site-level measurements for emission quantification. The acute need for trustworthy emission quantification methods has driven the development of novel monitoring approaches and their evaluation^7^. At present, source-level quantification is frequently done with hi-flow samplers^8^, which can give reasonably accurate results for known leaks which are easily accessible; however, hi-flow samplers are more challenging to use for high vent stacks and for leaks in equipment that are hard to reach for a human operator. Site-level quantification is typically done with airborne methane measurements (e.g. on remotely piloted aircraft systems or on manned aircraft). Liu et al.^9^ note that the errors in quantification can be considerable: although many systems can quantify “within an order of magnitude of the controlled release rate”, the absolute errors reported varied between 19 and 239%. The two remotely piloted aircraft systems (drones) tested even reported results with errors of 140% and 239%.

Another downside of all these periodically deployed technologies, both for source-level and site-level quantification, is that the results, at best, reflect the situation at the moment the survey took place. With periodic surveys alone it is not possible to accurately capture time-varying emission sources, or to demonstrate that large emissions are absent in the time interval between surveys.

Continuous monitoring offers further opportunity to improve methane emission management beyond periodic surveys^10^. Continuous monitoring can refer to source-level measurements, such as flow and composition meters in vents and other known emission sources, commonly referred to as Continuous Emissions Monitoring Systems (CEMS). It can also refer to continuous measurement of methane emissions from an entire facility, regardless of the source location. The present article reports on a first facility-scale deployment addressing the latter. Data from such a site-level continuous monitoring system are used for two purposes: (1) Real-time warning for early detection and indicative localization of unexpected sources; (2) Site-level quantification of emissions over longer time periods. The two purposes require separate data processing algorithms, whilst making use of the same set of measured data.

Site-level continuous emissions monitoring comes with inherent technical challenges. For instance, the performance of fixed point monitoring depends on the placement of the stationary receptors with respect to emission sources and local atmospheric conditions. A recent test with fixed point devices^10^ revealed that although these systems are capable of identifying large emissions occasionally, the true positive detection rate was smaller than with conventional handheld leak detection methods. Moreover, as reported in Riddick et al.^8^, the data analysis tools for fixed point receptors often make the assumption that the location of the emission point is known. This may be the case in simple gas release tests, but the information is usually not a priori available in actual deployments at oil and gas facilities where characterization of fugitive emissions is sought. Long open-path beam sensors, on the other hand, have been shown to be able to quantify total emissions from facilities fairly accurately^11,12^, but the commercial implementations have been focused at quantification of total emissions from sites or basins with a single instrument, with limited capability to detect, locate, and quantify unexpected single point emissions quickly.

In the present paper, we report the results from the first long-term industrial deployment of a multi-open-path laser dispersion sensor at an operating oil and gas facility. The deployment was organized after successful results from earlier field tests in idealized conditions^13,14^ validating both the instrument and the data analysis. We evaluate emission localization and quantification in an operational context, which differs from previous ideal cases in terms of (1) measurement duration, with the associated environmental and weather variations brought by 24/7 operation, (2) area covered, which is about 5-fold larger, and (3) topographic complexity owing to the elevated structures and potential elevated sources. The facility under scrutiny is a typical mid-size processing and distribution plant, located in The Netherlands. Methane concentration data was collected by a multi-open-path CH_4_ sensor over a period of three months. This data was combined with simultaneous wind measurements, and fed to a Bayesian data analysis algorithm to detect, locate and quantify methane emission sources. Two different algorithms were tested: one based on a fixed source grid, and one using a reversible jump scheme. The performance of the end-to-end monitoring system was first tested with a series of short-term controlled releases of process gas, followed by an analysis of long-term measurement data to detect, locate and quantify persistent methane sources. The results obtained with the unexpected emissions were compared with the outcome of a handheld OGI camera survey.

## Methods

### Multi-open-path laser spectrometer

Methane emissions from the facility are derived from temporal measurements of path-integrated CH_4_ concentrations across multiple directions. The optical sensor used is the Orion^TM^ Multi-Open-Path (MOP) middle infrared laser absorption/dispersion spectrometer from MIRICO Ltd. This type of laser spectrometer measures both the amplitude and the phase of light, leading to the recovery of, not only the molecular absorption, but also the dispersion^15^. The benefits of recovering the molecular dispersion spectrum as far as CH_4_ Path Averaged Concentration (PAC) measurements are concerned include improved resilience to light intensity fluctuations, linearity to gas concentration, and a large dynamic range^16^.

The Orion^TM^ MOP sensor radiates eye-safe beams to 5 cm aperture corner cube retro-reflectors up to few hundreds of meters away. The transmission optics is articulated in azimuth angles from 0° to 360°, and in elevation angles from −10° to +10°. This allows a measurement schedule to be programmed to sequentially measure a user-specified MOP pattern. The measurement precision is outputted by the instrument for each measurement. It is estimated from a global spectral fit approach^17^, which also provides measurements of water vapor from a neighboring molecular line. The generic precision of the CH_4_ concentration measurement is quoted to be 1 ppm $\times$ m, as 1 sigma precision normalized to the distance between the sensor and the retro-reflector; the “Concentration and meteorology data” subsection provides actual figures obtained during the deployment at the oil and gas facility.

The instrument used in the survey was the first version (Mk I), which occupies a volume of 85·90·100 cm^3^. To the sensor an additional large external chiller was added to supply a flow of temperature controlled water within the instrument, allowing it to cope with a wide range of ambient temperatures; all together the system weighted about 300 kg. The sensing system comes with a separate purpose-built meteorological station that measures pressure (based on barometric sensor HP206C from Hoperf), temperature (type K thermocouple), and the 3D wind velocity vector at a rate up to 10 Hz (Gill Instruments). From the high frequency wind velocity vector, turbulence statistics can be derived for each measurement cycle and inform the gas dispersion model. A cycle consists of the full MOP pattern measurement; it lasts 80 s. Each path measurement results from 4000 spectra averaged, with an individual spectra acquisition time of 0.8 ms.

Simple simulation studies were carried out before the deployment to determine the performances offered by different Orion^TM^ and retro-reflector placements. Based on historical wind data from a weather station close to the site, placing the Orion^TM^ in the North-East corner of the site and evenly spreading the retro-reflectors around the site boundaries maximizes the chance of plume intersection in prevailing wind conditions for sources almost anywhere at the site. Taking account of the need for fixed structures to support the equipment, the Orion^TM^ was placed on the office building, and the retro-reflectors were appended to lamp-posts around the site perimeter: the distribution of these structures was deemed to be close to the best placement identified by the simulations.

### Bayesian inference

High-precision MOP measurements of PAC of CH_4_ over time, contain information of background concentrations variations but also, more importantly, on possible gas emissions and transport across the wide area covered by the multi-beam pattern and beyond. Spatial and temporal concentration variations are coupled to emissions through the physics of gas dispersion (advection and diffusion). The wind vector diversity in magnitude and direction over time, together with the multi-directional MOP, is ideal to constrain the inference of emission locations and quantification from continuous concentration measurements^13^. The benefits of PAC measurements include a higher chance to intercept the plume and a more representative measurement than a point location one, due to the spatial averaging stemming from the path integration.

Bayesian inference^18^ combining physical modelling and statistical evaluation of the most likely solution is used to determine gas emission locations and rates, given the set of observed concentrations across the MOP pattern^14^. In principle the Bayesian inference method can be applied to any MOP implementation provided the PAC measurements are of sufficiently high precision, but in this work we apply it to measurements by the Orion^TM^ spectrometer from MIRICO Ltd. The posterior distribution, depicting our updated knowledge of source locations and corresponding emission rates, is derived from the likelihood function (using a gas transport model) and parameter prior distributions that describe our knowledge before any measurements are taken^19^. The posterior distribution is explored using a Markov Chain Monte Carlo (MCMC) algorithm, obviating the need for an analytical expression of the posterior. The gas dispersion model is the Draxler Gaussian plume^20^, with all input parameters derived from the high-frequency sonic anemometer data. The inference method and its theoretical basis has already been thoroughly described in the context of a first demonstration^13^ and subsequent blind trials^14^. Two approaches were used and compared for the Bayesian solver: fixed grid and reversible jump^21^.

In the case of fixed grid inversion, the entire area of the facility is considered and subdivided into contiguous square cells. The mass emission rates of all cells, assumed at ground level, is explored by the MCMC sampling to determine a solution that best matches the observed PAC data. In each cell, an identical ‘slab and spike’ prior distribution is assumed - this is a mixture distribution with two (truncated) Gaussians as components. The majority of grid locations are expected to be in the ‘spike’ component (with emissions close to zero) and a small number of sources in the ‘slab’ component (with larger emissions): this imposes the expected sparsity on the solution. More detailed information has already been reported^14^. Using a fixed grid is straightforward and allows easy adaptation of the grid resolution to the spatial information contained in the MOP concentration data. However, it does not include possible prior knowledge on facility equipment and associated locations of likely gas emissions. The computational complexity of a gridded solution also increases substantially when a third dimension is introduced, making this approach impractical when source height must also be estimated.

As an alternative, we use a Reversible Jump MCMC (RJMCMC) approach^22^. In this case, the number of sources, their locations and their emission rates are all estimated as part of the inversion. At each iteration of the algorithm, either an additional source is proposed in a new random location (a ‘birth’ proposal), or an existing source is deleted from the solution (a ‘death’ proposal). The new proposed solution is then tested, and accepted with high probability if it results in an improvement to the fit of modelled concentrations to the measured ones. The exact acceptance probability is computed as the product of the ratio of posteriors before and after the proposed change in the solution, the ratio of probabilities of making the state transition in either direction, and the Jacobian of the transition. Separately, all source locations are also updated at each iteration of the chain using a random walk Metropolis-Hastings scheme—again, each random proposal for a new source location is tested to check whether it improves or degrades the fit (as measured by the posterior distribution), and the probability of acceptance is determined accordingly^23^. This way, the solver has the freedom to explore all possible locations on a site in three dimensions, without being constrained to a grid: this is particularly helpful in situations where sources can occur at height to avoid the need to impose a 3D grid and the associated computational cost, and avoids the need to pre-set allowed locations for sources. The sparsity constraint is imposed on the solution through a Poisson prior distribution on the total number of sources. More mathematical details on the implemented RJMCMC are given in the Supporting information.

### Facility and monitoring system installation

**Figure 1:**
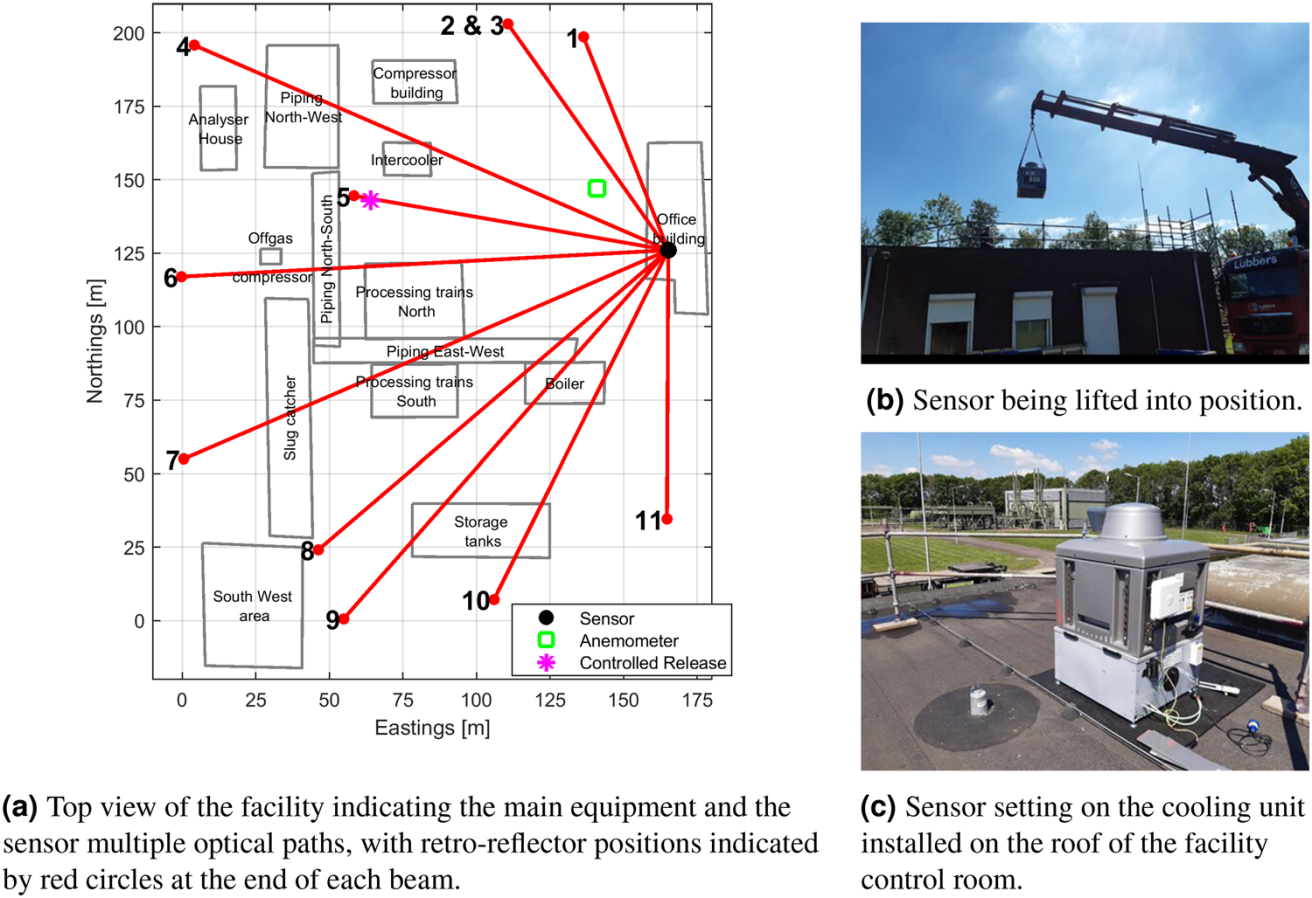
Settings and installation of the multi-open-path methane sensing system at the gas processing and distribution facility.

The processing and distribution facility under scrutiny is about 200·200 m^2^ (Fig. 1a). For the period of the survey (summer 2021) the daytime prevailing winds are South-Westerlies. Therefore, to maximize possible emissions to be captured by the optical beams, the instrument was positioned in the North East, at an elevated location (5 m), on top of the facility control room (Figs. 1b,c). At this height, the optical paths went over most of the facility equipment, unimpeded by people or vehicles on site. The facility is composed of various pieces of equipment, such as a dehydration unit, a compressor station, storage tanks, boilers, and piping racks. Any methane plumes emanating from these may thus be picked up as they disperse upward. Lamp posts around the facility were used to attach the distant retro-reflectors defining the beam paths. Eleven beam paths were formed into a fan using ten lamp posts as shown in Fig. 1a.

The path lengths ranged from 78.09 to 180.62 m. They were all horizontal at 5 m height, except for the beam 3 that was superimposed to beam 2, with a retro-reflector at 7.8 m. The higher reach of this upward slanted beam 3 aimed at increasing the chances to capture emissions from the 12 m high vent stack of the compressor building located at the Northern part of the facility. The meteorological station was also installed at 5 m onto a central lamp post. The purpose of the sonic anemometer part of the station is to measure the representative wind field for modelling methane transport from sources to optical beams. The location was chosen to minimize flow perturbation from neighboring structures for the prevailing winds. The anemometer was aligned to true north to a 1° precision. Optical sensor and retro-reflector locations were mapped to a 2 cm precision using a land surveying differential GPS module. The measurement schedule programmed was to sequentially measure each open-path from 1 to 11, staring for 5.4 s for each measurement, and home back to open-path 1 to finish the cycle. A full cycle lasted 80 s. Wind data were recorded as 1 s average, in order to capture fluctuations that are relevant for the atmospheric transport of methane plumes.

As part of the three month continuous survey, transient controlled releases of methane were organized to evaluate and calibrate the emission monitoring approach. For this purpose, a needle valve was connected to the low-pressure gas line leading to the compressor station. The composition of the gas emitted in the controlled release was known from the data from the ‘analyser house’ on site, which provides the weight percentage of methane in the composition of the export gas in real time. A 20 m long hose terminated by a perforated ring (1.0 m diameter) was attached to the valve to mimic a methane release. A flow-meter (Brooks Instrument GT1000) was used to measure rates from 4.7 and 5 kg/h when the needle valve was left half-open. The ring was placed in the empty area south of the compressor building, indicated by the magenta star in Fig. 1a. More details on the controlled release set-up are provided in the Supporting information.

## Results

### Concentration and meteorology data

**Figure 2:**
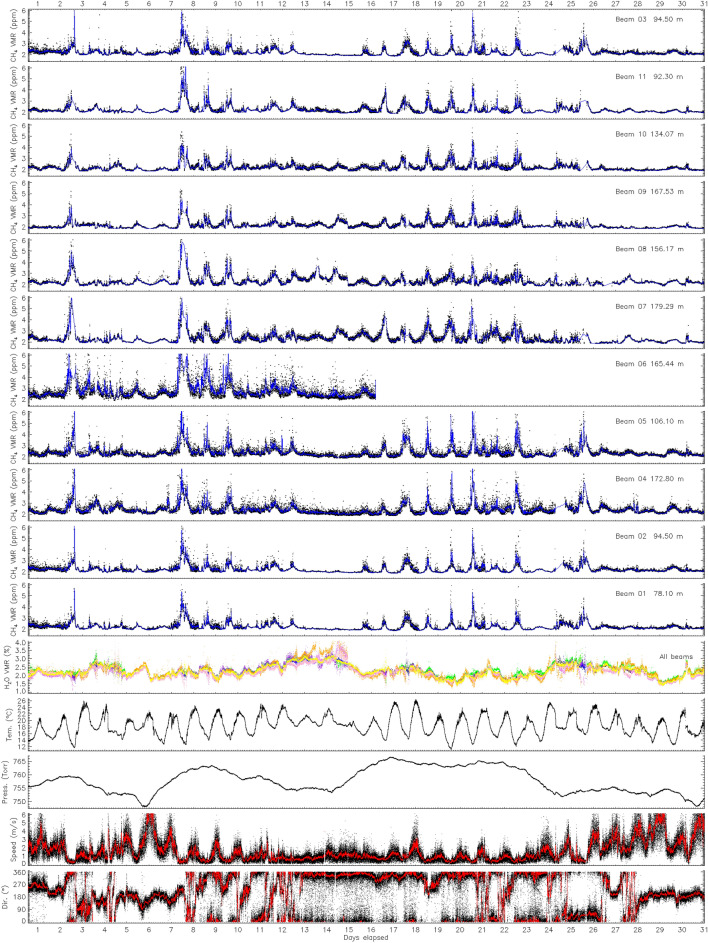
Subset of continuous measurements over the month of July 2021 of multi-path averaged concentrations of methane and associated meteorological data recorded by the sensor. The time ticks on the abscissa correspond to UTC noon for each day. The blue (red) line corresponds to a 10 (60) point smoothing.

The data collected can be represented as temporal series of continuously measured parameters for three months. Instrument uptime was 99.8%, data collection only stopped during brief heavy rain showers caused by reduction of returned optical signal beyond ~4 orders of magnitude; signal returned quickly when the rain became less intense. Figure 2 shows an excerpt covering July 2021 data for eleven CH_4_ PACs, water vapour PACs, external temperature and pressure, and wind velocity and direction. The full campaign data plots are provided in the Supporting information document. Beam 6 measurements were interrupted mid-July due to a stork displacing the corresponding retro-reflector; due to the high position of the retro-reflector, it could not be repaired on short notice. The precision of the concentration data as estimated from the raw instrument noise was 4 ppb on the PAC (one sigma). Total random noise on the measured PACs ranged from 10 to 20 ppb, scaling with path length. The estimation is done by calculating the standard deviation of PACs over a 9 h “quiet” period over which the detrended CH_4_ concentrations are stable (high wind episode around noon on the 6th of July, more details in the Supporting information). No drift was observed in the methane concentration data during the three months of the trial. The daytime background concentration observed at the site is generally between 1.9 and 2.2 ppm. The instrument was measured to drift up to 1% over 24 h before installation. No further calibration was performed during the campaign as the analysis can account for systematic biases, included in the model.

The data series in Fig. 2 already brings interesting aspects related to the continuous monitoring of an oil and gas facility. Early on, PACs measured over beam 6 were an outlier as they were both higher and more variable. This suggested a small source of methane in the close vicinity of the path. The findings described in the Persistent sources later confirmed this early suspicion. The July 2021 data also shows daily modulation of concentrations, which can sometimes peak well above 10 ppm in the middle of the night, particularly for beam 6 due to the suspected local source. These spikes correspond to a nocturnal inversion layer trapping diffuse CH_4_ emissions^24^ and have little relation to the facility operation, except for the PACs over beam 6, which again shows spikes of higher concentrations compared to other PACs. The prevalent atmospheric conditions are therefore critical to consider when setting CH_4_ detection alarm^8^. Alarms based on simple threshold of absolute concentration levels are bound to fail.

### Controlled gas releases

Four controlled releases, lasting between 45 and 60 min, were carried out during the survey: three on the 28th of July and one on the 3rd of August 2021. The locations and the mass emission rate used were known to the analysis team. Data from all four controlled releases was combined into a single run of the inversion procedure.

**Figure 3:**
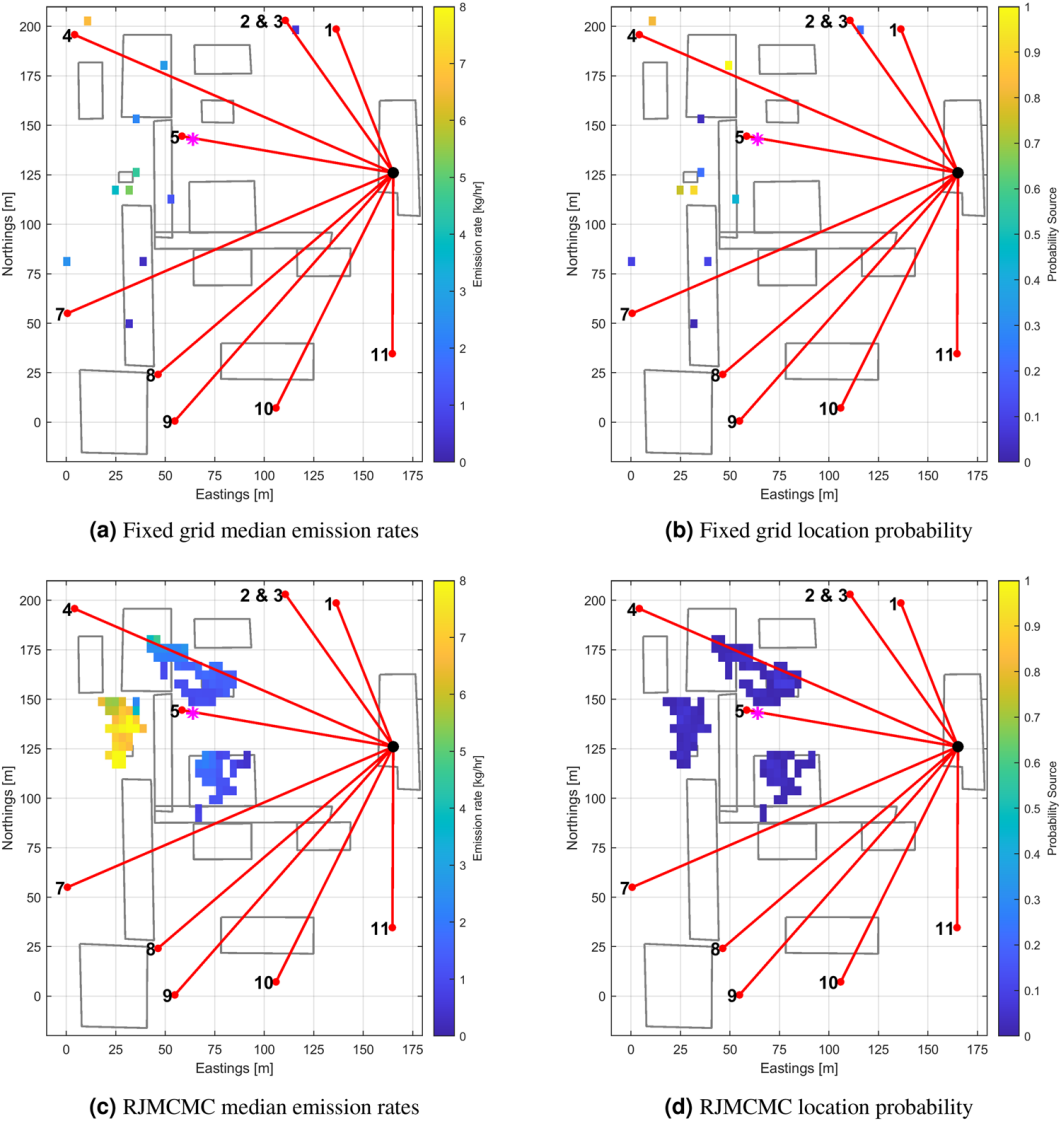
Maps of posterior median mass emission rates and associated location probabilities inferred from the data recorded during the controlled release events on July the 28-th and August the 3-rd 2021. The true location of the release is indicated by a magenta star. More information about the analysis is provided in section 4 of the Supporting information document.

The fixed grid inversion was performed using an approach already reported^14^, where a slab and spike distribution was used for the source prior. A 50 by 50 grid of cells was imposed on the site, giving a grid size of 3.5 m by 4.5 m. The spike probability was set to 99% of the total grid cells, with a standard deviation of 0.001 kg/h as we expect most of the fixed grid not to emit any methane (this neglects the comparatively small persistent emisisons from leak sources that were present on the site). The complementary slab standard deviation was set to 4 kg/h to allow solution exploration up to about 16 kg/h. Figure 3a shows the posterior source map (median emission rate for each grid cell) after 5000 MCMC iterations. Figure 3b shows the corresponding posterior location probability map. For all the emission rate maps reported, only the sources greater than 0.001 kg/h and/or with a probability greater than 0.01 have been retrained. The MOP are represented as red lines; the true source location is indicated by the magenta star directly under the position of retro-reflector of beam 5. There are four sources in the source map which are present for more than 50% of the iterations in the solution:

- A source directly under beam 4 with conditional median emission rate 2.8 kg/h, and 1-sigma range of 0.6 kg/h. As an analogy to the well known case of a Gaussian distribution, unless stated otherwise uncertainties are reported as 1-sigma ranges, calculated as half of the 16–84% quantile ranges of the posterior distribution. However, the posterior distributions are not Gaussian.
- Two sources close together at 30 m easting, 120 m northing with a conditional median emission rates of 3.8 and 5.3 kg/h, and 1-sigma ranges of 2.6 and 2.9 kg/h respectively.
- A source at the north-western edge of the domain. This source has 1-sigma range of 5.8 kg/h, but a wide probability distribution (>20 kg/h for the 2.5–97.5% quantile range, c.f. Supporting information). It is considered to be a boundary artifact which only contributes to explaining a small number of data points, since the observed winds blew from the South and East.

The solution from the actual controlled release is likely to correspond to the 5.3 ± 2.9 kg/h source, whilst the two additional lesser sources are added to explain elevated concentrations due to persistent sources in the vicinity of beam 7 (to be covered in “Persistent sources”). Precise attribution is difficult given the unexpected interfering persistent emissions close to the release site, and the limited length of the controlled release data set. The loss of data from beam 6, and the reduced wind diversity during these short controlled releases (meaning only a subset of the beam fan “saw” the plume) results in a location determination poorly constrained. Retaining only the most intense source recovered from the inversion, the mass emission rate quantification is very close to the actual. The inferred location is however 30 m away from the actual one. The error in the localisation is mainly in the direction of the sensor, which is in general the direction with the highest localisation uncertainty due to the geometry of the beam layout. Especially since there was little wind variability during the controlled release events, localisation turned out to be challenging. In addition to a poorly constrained inversion, gas dispersion model errors also contribute, as a free flow Gaussian model is used whilst topographic obstacles at the facility affect gas flow. The measurements were made with predominant southerlies pushing the plume against a 10 m height compressor station. The current dispersion model does not capture such effects^25^.

Using the same measurements, inversion with the RJMCMC was performed. The resulting source and probability maps are given in Fig. 3c,d, respectively. Three distinct clusters of potential source locations were inferred. Using RJMCMC, solutions for sources can occur freely in 3D space, so a grid is imposed in order to bin and summarize the results: the location probability is obtained by calculating the proportion of the MCMC iterations for which a cell contained at least one source; summary statistics (medians and uncertainty ranges) are then calculated using only those iterations for which a source was present in the cell (i.e. conditionally on the presence of a source, ignoring iterations for which the cell is empty). No individual location is persistent throughout all posterior distribution samples. A cluster of locations appearing in the solution is indicative that a source is likely present within that area, with the spatial extent of the cluster giving information about the uncertainty in the source location. The posterior probability map (Fig. 3d) indicates the probability of the different locations within the cluster, the conditional median map (Fig. 3c) indicates the median strength of a source *if* it lies within that grid cell. The average emission rate for the source corresponding to a given cluster is therefore a weighted average of all the conditional average emission rates for cells in the cluster. The resulting outcome bears some similarity to the fixed grid solution in terms of location. In the most intense cluster, the individual cells have median emission rates between 3 and 8 kg/h, with an overall cluster median of 7.4 kg/h. The uncertainties (still expressed as 1-sigma values, being half the 16–84% quantile ranges) for the individual cells span ± 0.8 to ± 2.5 kg/h, with the 1-sigma range for the whole cluster being 1.5 kg/h.

### System gas release

**Figure 4:**
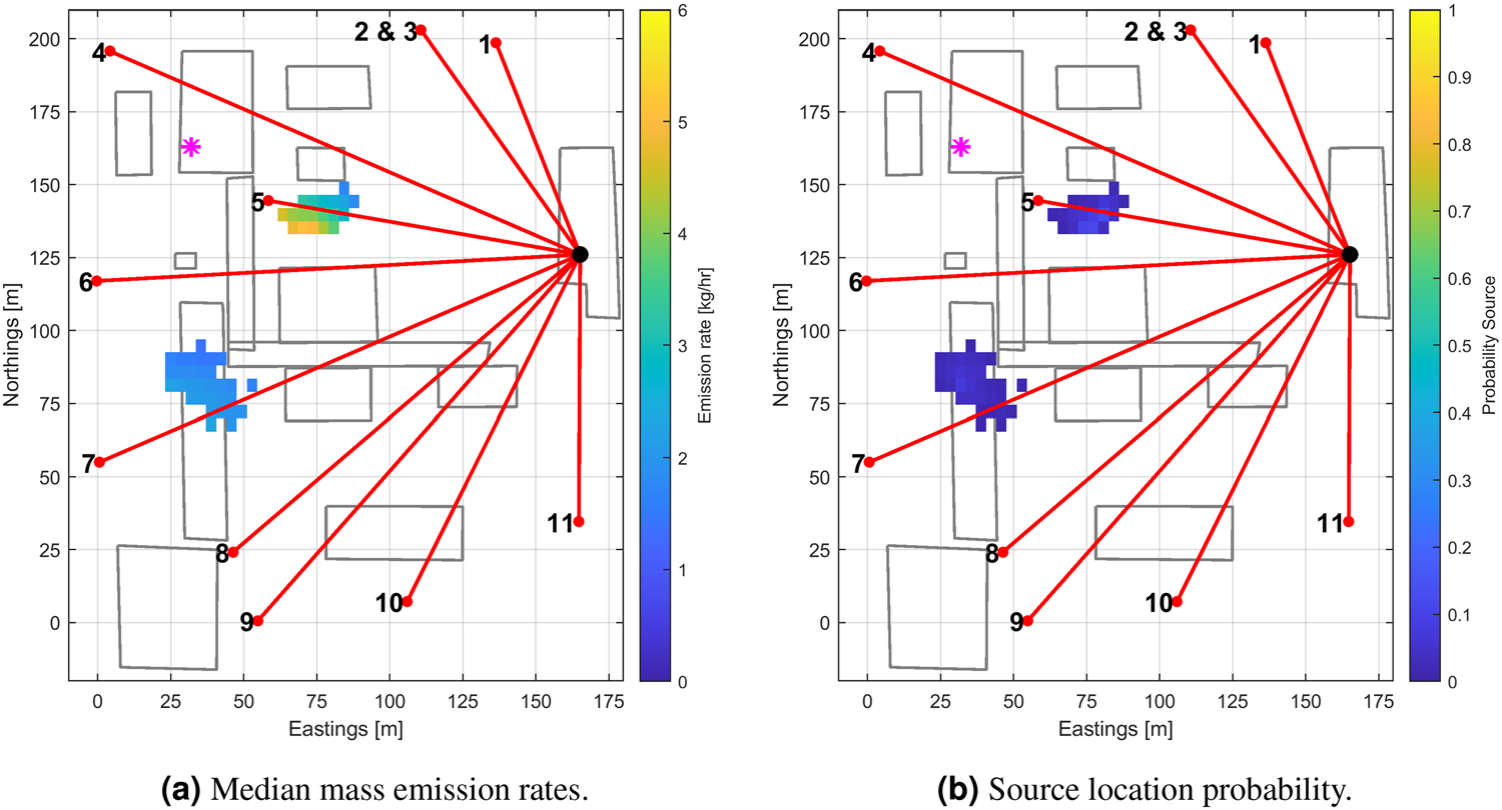
Output posterior maps (**a**) and associated location probability (**b**) inferred from a reversible jump MCMC analysis of the data recorded during the system release event on July the 7-th 2021. The true location of the release is indicated by a magenta star. More data on the analysis is provided in the section 5 of the Supporting information document.

As part of the facility operation, one maintenance system release occurred on 7 July 2021, for three hours. The data analysis team was made aware of the event, but not of the associated location and mass emission rate. Recorded data were analysed using RJMCMC, with parameter settings identical to the controlled release case. RJMCMC analysis was preferred as it allows free exploration of all possible source locations in 3D space. The results are shown in Fig. 4 in which both the posterior median emission rate map and the associated location probability map are given. Two main clusters of locations with non-zero probability of a source are determined (Fig. 4b); the cluster further north was selected as the most trusted inferred location based on (1) the high location probability, (2) the strong concentration signal observed on beam 4, and (3) the fact that this location never appeared in control runs made outside any known intentional release. The location retained is also the strongest emitter: the conditional median mass emission rates for the individual cells are between 2 and 5 kg/h, as shown in Fig. 4a. The cluster as a whole has a median mass emission rate of 3.7 kg/h, and a 1-sigma uncertainty range of 1.1 kg/h. After the analysis, the actual location was reveal and is indicated by a purple dot in Fig. 4a. The actual emission rate for the system release was not known. The most probable cluster inferred corresponding to the release is about 40 m away from the actual position; the discrepancy is likely to originate from the same causes indicated for the previous case on controlled releases. The second cluster is believed not to be a false alarm, but again an indication of the presence of persistent sources to be presented in the next section.

### Persistent sources

As mentioned in the “Concentration and meteorology data” subsection, from the temporal concentration traces, persistent, low intensity, emissions from parts of the facility were already suspected. Controlled and system release inferences also revealed additional unexpected sources. Inversion runs using both the fixed grid and the reversible jump approaches were conducted on data outside any planned releases. A period of 30 h spanning the 6th and 7th of July 2021, which contains more than 24 h of data with large varying wind directions was selected as input for the analysis.

For the fixed grid inversion, a refinement was added: the grid was only considered in areas coinciding with facility equipment as emissions are not expected to come from elsewhere. The grid was 50 by 50 cells, the full facility equipment was covered by 2500 cells. In contrast to the fixed grid case presented for controlled releases, the height of the equipment was also used to constrain grid elements. For the reversible jump inversion, the settings were left unchanged.

**Figure 5:**
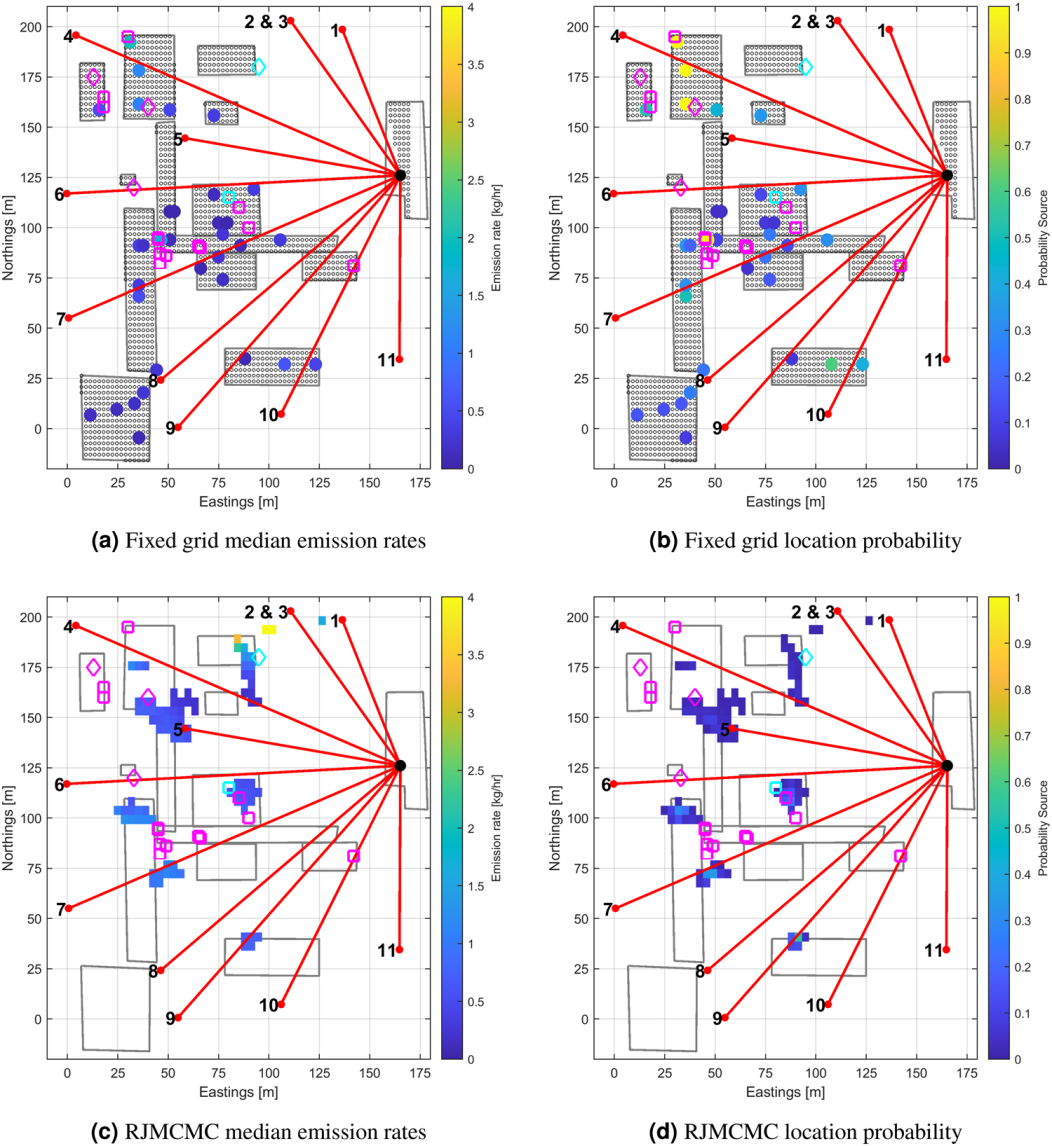
Maps of posterior median mass emission rates and associated location probabilities inferred from the data recorded outside any release event revealing the presence of low intensity persistent emissions from the site. The magenta and cyan square and diamond symbols indicates the location of emission sources subsequently confirmed using a gas camera: the cyan markers indicate the sources that were believed to be the most intense.

The resulting maps of median mass emission rates and location probabilities for both cases are shown in Fig. 5. The fixed grid inversion (Fig. 5a,b) highlights a number of areas with potential emissions. The reversible jump inversion, shown in Fig. 5c,d, is not constrained to equipment locations but it still shows consistency with the fixed grid inversion, both in terms of locations and emission rates (mostly sources of about 1 kg/h). A notable exception are the high emission rates (up to a median of 4 kg/h) north of the compressor station - these are isolated clusters associated with a relatively low total probability of emission.

**Figure 6:**
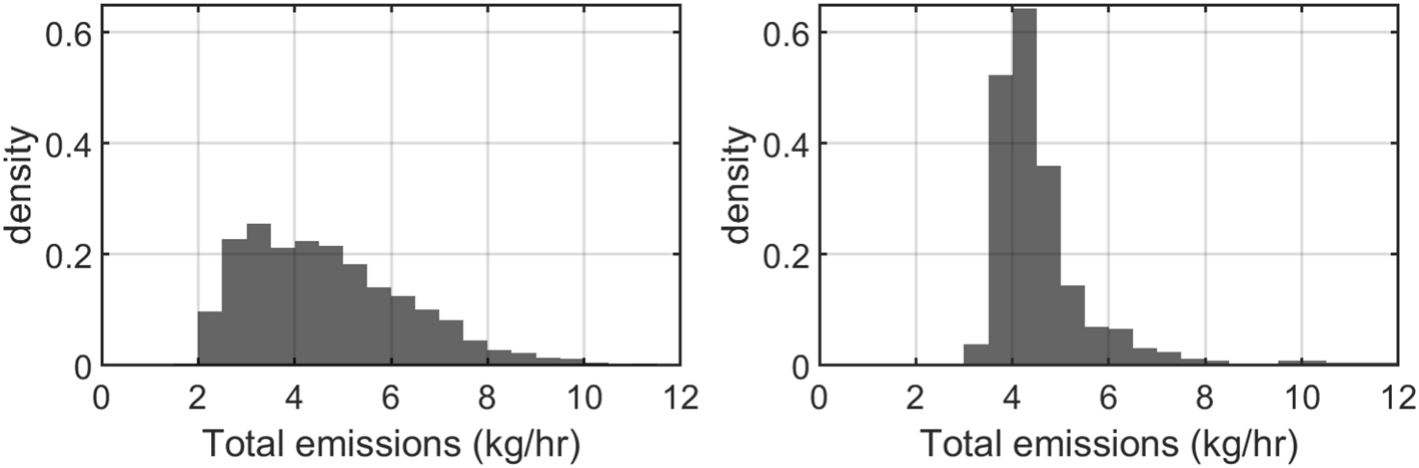
Probability density functions of the total mass emission rate from the site. Left, fixed grid case; right, reversible jump case.

Given the scatter of multiple small emission sources suggested by the inversions, the posterior distribution of the total emission from the site was estimated. The site total emissions for each MCMC iteration are calculated by simply summing the contributions of the individual sources present. Figure 6 shows the probability density functions as predicted by the MCMC algorithm in thousands of iterations. The median emission rate is 4.5 kg/h for the fixed grid inversion, and 4.3 kg/h for the reversible jump algorithm. Compared to the fixed grid case, the distribution in the reversible jump case is more peaked around the mean value: the 1-sigma quantile range for the fixed grid case is 1.8 kg/h, whereas for the RJ case it is 0.8 kg/h. The probability density function also has a longer tail, related to the highly uncertain estimate of the large emission from the vent stack of the compressor building. In general, however, the estimate of the most likely emission rates is consistent between the two methods.

By definition, the quiet persistent emissions from the facility were not controlled. To corroborate the findings from the continuous monitoring, a survey of the site using an oil and gas industrial (OGI) camera (FLIR GF320) was conducted on the 3rd and 11th of August. The condensate tanks at the south of the site were not covered due to lack of survey time. Eighteen leaks were identified from the survey, and are mapped in Fig. 5 as magenta and cyan squares and diamonds. Diamonds correspond to vent points. Locations verified agree reasonably well with the inferred maps in terms of cluster of sources. The spatial resolution of the inferences was not sufficient to pinpoint individual sources, but identified the equipment coarse location instead. The OGI camera does not produce emission rate estimates that can be used to compare with the results from the MOP continuous monitoring. Instead, a qualitative relative ranking was performed by the operator based on visual feedback. The two sources identified in Fig. 5 by cyan markers were thought to be the most intense.

## Discussion

Results on controlled releases suggest that emissions of the order of 5 kg/h could be unambiguously detected by the system within a period of 45–60 min. No single release event was completely missed. Spurious emissions were also returned by the analysis, and were traced back to unexpected persistent emissions distributed over the facility. Compared to the ideal case of a flat open field^14^ where a relative quantification error of 30% was obtained, emission quantification was degraded up to 50%, impeded by the facility topography, and the presence of quiet persistent sources. Biases of approximately 30 m on the location were observed. In the context of emission quantification within oil and gas facilities, where quantification error of 100–200% are common^7,9^, the results obtained are promising, particularly for low level emissions (<10 kg/h). Overall the RJMCMC samples the solution space more thoroughly than the fixed grid MCMC and tends to be preferred to provide equipment locations requiring scrutiny. To account for disruption to the gas flow field generated by facility obstacles and reduce bias, adaptation of the model to include building downwash^26^ could be envisioned for future work.

The unexpected presence of low intensity persistent emissions from the site has allowed demonstration in a real test case. Since these sources were persistent, the analysis could be applied to measurement data acquired over longer periods of time, with a broader variety of wind conditions. A few anomalies were detected, and clusters of leaky areas were clearly identified and quantified, despite low emission rates (< 1 kg/h). Eighty percent of the persistent sources found by a subsequent OGI survey were successfully identified. In the southernmost region not surveyed by the OGI camera, the consistency between fixed grid and RJMCMC results on a high probability source occurring at the storage tank suggests this source is real. The confidence in the sources identified by the fixed grid inversion procedure in the South West area is much lower. From the inference an estimate of the total emission rate of the facility was derived, with good consistency regardless of the MCMC method used. A high-level summary of the results obtained throughout the deployment is provided in Table 1.

**Table 1:** Summary of quantification and localization results of the methane emissions across the site for all scenarios considered during the deployment.

MCMC method	Inferred source			Actual sources
	Median emission rate (kg/h)	Uncertainty (kg/h)	Distance to actual (m)	Emission rate (kg/h)
Controlled release				
Fixed grid	5.3	2.9	30	4.9 ± 0.1
Reversible jump	7.4	1.5	30–50	4.9 ± 0.1
System release				
Fixed grid	N/A	N/A	N/A	N/A
Reversible jump	3.7	1.1	40	Unknown
Total emissions from persistent sources				
Fixed grid	4.5	1.8	10	Unknown
Reversible jump	4.3	0.8	20	Unknown

The results from this first deployment are encouraging in view of more mature installations of the technology in the future. The fan of eleven beams was found resilient (loss of one beam) with minimal tampering with the facility equipment. The remote nature of the measurement ensures installation outside ATEX zones as far as on-ground facilities are concerned. Continuous monitoring systems have potential advantages over currently-used technologies for source-level and site-level methane quantification. Firstly, the ability to detect leaks early. Based on the results obtained during the present deployment, we estimate that it must be possible to reliably detect methane emissions of the order of 5 kg/h from a facility of a similar size used here (about 200 m by 200 m in surface area). This will require the development of a “decision” algorithm analysing inferred data and their confidence metrics into a detection event. Secondly, the system can quantify over long periods of time, allowing for variable emissions to be characterized as time series to validate the impact of intervention. Reciprocally, the present system can be used to demonstrate absence of large emissions during a prolonged measurement period.

This trial also highlighted some of the challenges of using the new system for continuous monitoring at an oil and gas facility, and pointed to areas for future work. The data analysis procedure used relies on a Gaussian plume model, which is known to be unsuitable in cases of low wind speeds and does not account for obstacles in the flow field: future development should adapt the gas dispersion model to augment the exploitation of the available data, particularly during overnight temperature inversions. The analysis procedure should also be adapted to allow simultaneous or sequential analysis of multiple data segments, to constrain the locations of persistent site emissions better: to achieve this, emission rates can no longer be assumed constant in time, owing to changes in site operations, and the analysis model will need to account for this.

## Conclusion

This paper describes the first-ever 3-month deployment at an operational mid-stream oil and gas facility of a continuous, wide area, monitoring system consisting of the combination of a multi-open-path high-precision methane concentration analyser (<10 ppb) and a data analysis method with Bayesian inference. The performance of the technology has been very encouraging. Controlled short-term gas releases of 5 kg/h were unambiguously picked up and quantified reasonably well, with an emission rate of 5.3 ± 2.9 kg/h in the fixed grid inversion and 7.4 ± 1.5 kg/h for the RJ inversion, showing efficient detection and quantification of emissions of approximately 1 h in duration. Interestingly, the long-term continuous measurements clearly establish the impossibility of relying of a simple methane concentration threshold for accurate detection, owing to the variations of atmospheric conditions. The localization of sources of emissions was found to be approximately three times less accurate than earlier experiments in an open flat field for which the free flow Gaussian plume model is adequate to describe gas dispersion. In the case of a oil and gas facility, flow obstruction and complex topography reduces the validity of the gas dispersion model; ideally, the gas dispersion model should include information on the site layout. The coincidental finding of unexpected persistent sources across the facility has exemplified the value of the novel monitoring system. The continuous monitoring system, based on the plume triangulation stemming from wind diversity and enabled by the multi-open-path geometry, has demonstrated the capability to unveil weak sources with confidence. As a result, targeted, efficient repair efforts can be conducted. The capability to report the total emissions from site was also developed and demonstrated.

### Supplementary Information

Supplementary Information.

## Data Availability

The datasets generated during and/or analysed during the current study are available in the STFC eData repository [10.5286/edata/919].

## References

[1] IPCC. *Climate Change 2013: The Physical Science Basis. Contribution of Working Group I to the Fifth Assessment Report of the Intergovernmental Panel on Climate Change* (Cambridge University Press, Cambridge, United Kingdom and New York, NY, USA, 2013).

[2] Nisbet EG (2019). Very strong atmospheric methane growth in the 4 years 2014–2017: Implications for the Paris agreement. Glob. Biogeochem. Cycles.

[3] Saunois M (2020). The global methane budget 2000–2017. Earth Syst. Sci. Data.

[4] Peng S (2022). Wetland emission and atmospheric sink changes explain methane growth in 2020. Nature.

[5] United Nations Environment Programme and Climate and Clean Air Coalition. *Global Methane Assessment Benefits and Costs of Mitigating Methane Emissions* (2021).

[6] United Nations Environment Programme. Mineral methane initiative OGMP2.0 framework. Tech. Rep. (2020).

[7] Ravikumar AP (2019). Single-blind inter-comparison of methane detection technologies–results from the stanford/edf mobile monitoring challenge. Elementa Sci. Anthropocene..

[8] Riddick SN (2022). A quantitative comparison of methods used to measure smaller methane emissions typically observed from superannuated oil and gas infrastructure. Atmos. Meas. Tech..

[9] Liu Y (2023). Assessment of current methane emissions quantification techniques for natural gas midstream applications. Atmos. Meas. Tech. Discussions.

[10] Bell CS, Vaughn T, Zimmerle D (2020). Evaluation of next generation emission measurement technologies under repeatable test protocols. Elementa Sci. Anthropocene..

[11] Alden CB (2019). Single-blind quantification of natural gas leaks from 1 km distance using frequency combs. Environ. Sci. Technol..

[12] Wu C-F (2014). Measurement of fugitive volatile organic compound emissions from a petrochemical tank farm using open-path fourier transform infrared spectrometry. Atmos. Environ..

[13] Hirst B (2020). Methane emissions: Remote mapping and source quantification using an open-path laser dispersion spectrometer. Geophys. Res. Lett..

[14] Weidmann D (2022). Locating and quantifying methane emissions by inverse analysis of path-integrated concentration data using a markov-chain monte carlo approach. ACS Earth Space Chem..

[15] Wysocki G, Weidmann D (2010). Molecular dispersion spectroscopy for chemical sensing using chirped mid-infrared quantum cascade laser. Opt. Express.

[16] Daghestani NS, Brownsword R, Weidmann D (2014). Analysis and demonstration of atmospheric methane monitoring by mid-infrared open-path chirped laser dispersion spectroscopy. Opt. Express.

[17] Robinson I, Butcher HL, Macleod NA, Weidmann D (2019). Hollow waveguide integrated laser spectrometer for 13co2/12co2 analysis. Opt. Express.

[18] van de Schoot R (2021). Bayesian statistics and modelling. Nat. Rev. Methods Primers..

[19] Humphries R (2012). Atmospheric tomography: A bayesian inversion technique for determining the rate and location of fugitive emissions. Environ. Sci. Technol..

[20] Draxler R (1976). Determination of atmospheric diffusion parameters. Atmos. Environ..

[21] Hirst B, Jonathan P, GonzálezdelCueto F, Randell D, Kosut O (2013). Locating and quantifying gas emission sources using remotely obtained concentration data. Atmos. Environ..

[22] Green PJ (1995). Reversible jump Markov chain Monte Carlo computation and Bayesian model determination. Biometrika..

[23] Zanini E, Eastoe E, Jones MJ, Randell D, Jonathan P (2020). Flexible covariate representations for extremes. Environmetrics..

[24] Iriana W (2016). Measurement of carbon dioxide flux from tropical peatland in Indonesia using the nocturnal temperature-inversion trap method. Environ. Res. Lett..

[25] Stockie JM (2011). The mathematics of atmospheric dispersion modeling. SIAM Rev..

[26] Schulman LL, Strimaitis DG, Scire JS (2000). Development and evaluation of the prime plume rise and building downwash model. J. Air Waste Manag. Assoc..

