# Supplementary material for: Long-term continuous monitoring of methane emissions at an oil and gas facility using a multi-open-path laser dispersion spectrometer
Source: Sci Rep. 2024 Jan 5;14:623. doi: 10.1038/s41598-023-50081-9 (PMC10770390; doi:10.1038/s41598-023-50081-9)
Supplement: Supplementary file 1 — Supplementary Information. [file 41598_2023_50081_MOESM1_ESM.pdf]

# Supplementary Information

# Long-term Continuous Monitoring of Methane Emissions at an Oil and Gas Facility using a Multi-open-path Laser Dispersion Spectrometer

**Rutger IJzermans[1], Matthew Jones[1], Damien Weidmann[2,3,*], Bas van de Kerkhof[1], and David Randell[1]**

[1]Shell Global Solutions International B.V., Grasweg 31, 1031 HW Amsterdam, The Netherlands
[2]STFC Rutherford Appleton Laboratory, Space Science and Technology Department, Didcot, OX11 0QX, UK
[3]MIRICO Ltd, Unit 6, Zephir Building, Harwell Campus, Didcot, OX11 0RL, UK
[*]damien.weidmann@stfc.ac.uk

## 1 Controlled release experiment

Further pictorial information on the setting up of the controlled gas releases is provided in figure 1 and figure 2.

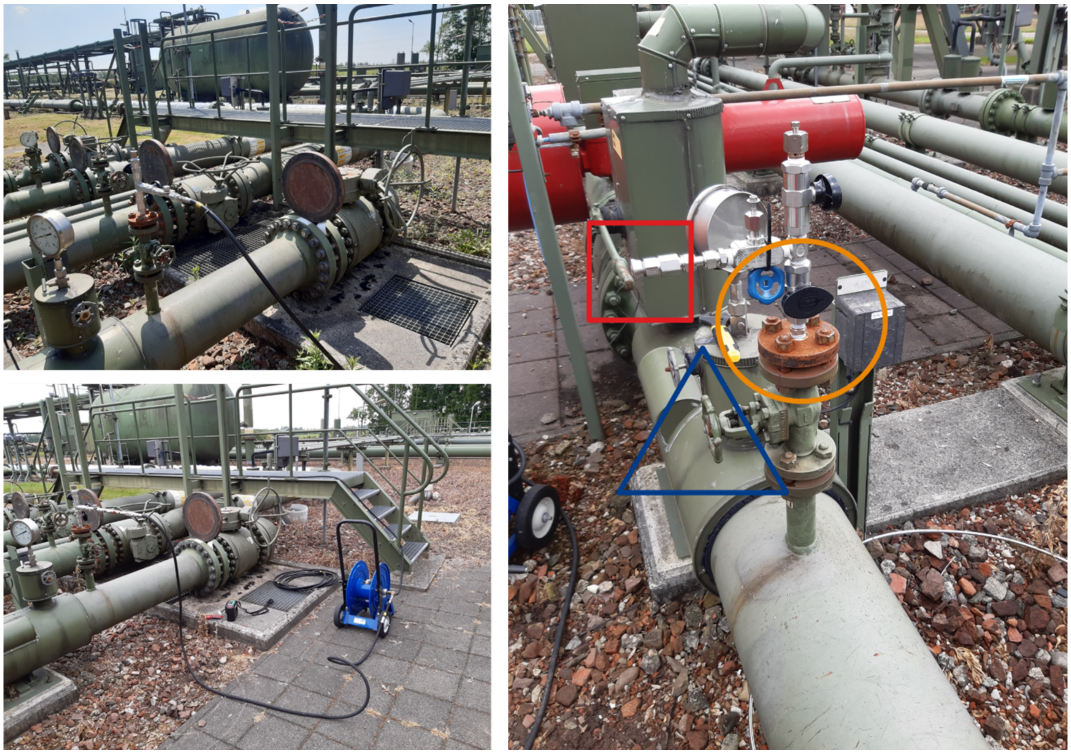

**Figure 1.** Controlled release functionality on the low-pressure gas line that goes to the compressor building. Top left: connection to the pipeline through the nitrogen purger. Bottom left: for a controlled release, the 20 m long hose is put across the walking bridge into the empty area towards the South of the compressor building. Right: the controlled release system when it is not operational. The red square shows the Swagelock connections to the hose; the orange circle shows the black valve, which is in closed position on the picture. The blue triangle shows the existing valve on the pipeline.

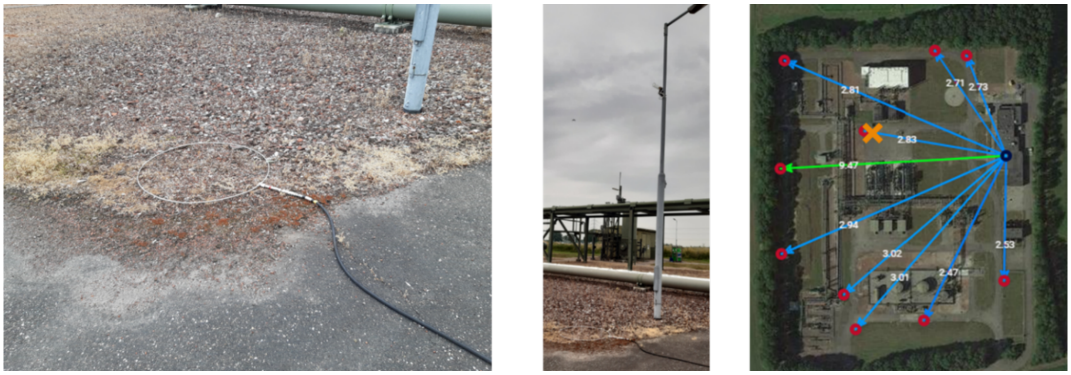


**Figure 2.** Location of perforated ring from which controlled release of methane is executed. Left: perforated ring; the hose is attached to the connection point on the low-pressure gas line as shown in figure 1. Middle: perforated ring under the lamp post with a retro-reflector attached. Right: top view of the site with open path beam layout; the orange cross indicates the controlled release point (background aerial picture from Google Earth).

## 2 Error estimation on path averaged concentration

The multi-open-path sensor reports for each measurement the precision on the $CH_4$ concentration, derived from the spectral fitting algorithm, and representing the instrument noise propagation to the concentration measurements. The distribution of instrument precision over the month of July is shown in figure 3.

The estimation of the full precision of the concentration measurements including all effects is estimated from detrended measurements across a period of time where the measurement time series is as stable as possible (limited atmospheric effects). An example of detrended dataset is shown in figure 4. With this approach the full error on PAC is estimated to be 2 times that reported by the spectral fitting algorithm solely taking into account detector noise.

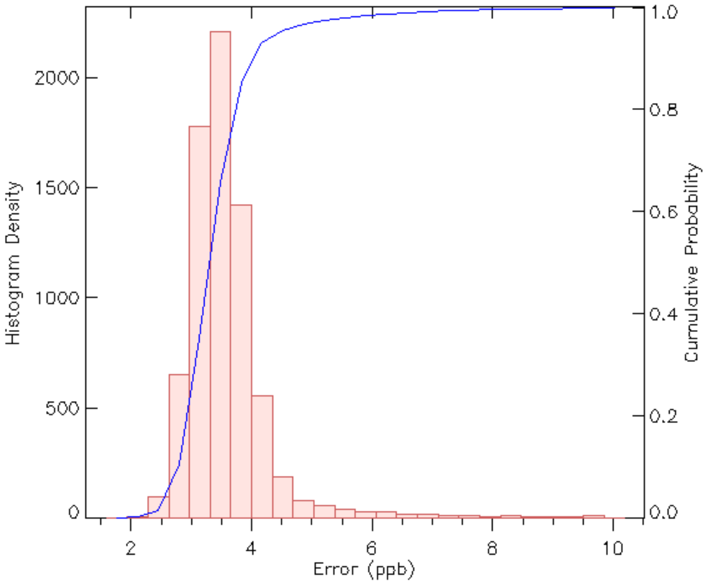


**Figure 3.** Histogram of the distribution of instrument $CH_4$ concentration error over all the measurements from the month of July 2021.

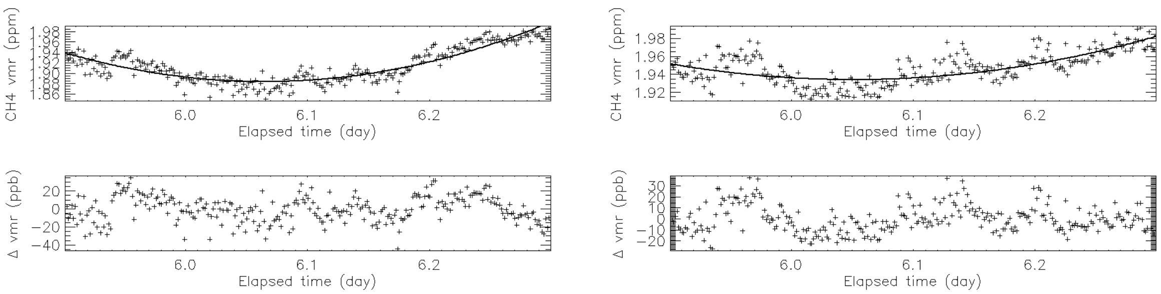


**Figure 4.** Example of estimation of the full error through detrending of a stable concentration record. The top plots show a stable subset of PAC data overlaid with the detrending polynomial. The resulting detrended residuals are shown in the second row.

## 3 Reversible Jump Markov Chain Monte Carlo algorithm

In this model, the number of sources, their locations and their emission rates are all treated as unknown random variables: a prior distribution is specified for each. The prior distribution for the source locations states that sources are equally likely to be located anywhere within the site footprint, between 0 and 5 metres above ground level. Prior distributions for the individual source emission rates are truncated Gaussians, so as to prevent negative emission rates. The other components of the model (background concentration time-series, sensor offsets, measurement error precision) are specified and estimated in the same way as outlined as in previous work[1]. Combining these priors with the priors for atmospheric background concentration and measurement error precision and the data likelihood gives the full joint posterior distribution given the observed concentrations. Up to normalization, the posterior is expressed by Eq.1.

$$p(\mathbf{s},\mathbf{Z},\mathbf{b},\lambda|\mathbf{y}) \propto p(\mathbf{y}|\mathbf{s},\mathbf{Z},\mathbf{b},\lambda)p(\mathbf{s}|\mathbf{Z},n)p(\mathbf{Z}|n)p(n)p(\mathbf{b})p(\lambda) \tag{1}$$

The terms appearing in Eq.1 are:

- $\mathbf{y}$ is the vector of observed concentration measurements.
- $\mathbf{s}$ is the vector of source emission rates.
- $\mathbf{Z}$ is the set of 3-dimensional source locations.
- $n$ is the number of sources.
- $\mathbf{b}$ is the vector of background concentration values.
- $\lambda$ is the set of measurement error precision parameters (one parameter per sensor).

As in the previously described fixed grid case[2], the data likelihood $p(\mathbf{y}|\mathbf{s},\mathbf{Z},\mathbf{b},\lambda)$ is assumed to be Gaussian, the background prior $p(\mathbf{b})$ is also set to be a Gaussian, and the prior for each measurement error precision term is set to be a gamma distribution.Because of the choices of priors conjugate to the likelihood, the conditional distributions for $\mathbf{b}$ and $\lambda$ can be derived and sampled from in closed-form.

Samples of the number of sources, source locations and source emission rates are generated from the posterior distribution during the MCMC chain. To sample solutions containing different numbers of sources, we use the reversible jump algorithm[3]: at each iteration, either a 'birth move' (adding one source to the solution) or a 'death move' (removing one source from the solution) is proposed, compared against the current solution, and accepted or rejected accordingly. The approach of[4] is broadly followed: denoting the current state of the MCMC chain by $\Omega = \{\mathbf{s},\mathbf{Z},n,\mathbf{b},\lambda\}$, the dimension-jumping proposal generates a new state $\Omega^* = \{\Omega \setminus \omega, \omega^*\}$: i.e. the parameter set $\omega = \{n,\mathbf{Z},\mathbf{s}\}$ is substituted for the augmented parameter set $\omega^* = \{n^*,\mathbf{Z}^*,\mathbf{s}^*\}$. The proposed state is then accepted with probability $p$ given by Eq.2.

$$p = \min\left(1, \frac{p(\Omega^*|\mathbf{y})}{p(\Omega|\mathbf{y})}\frac{q(\omega|\omega^*)}{q(\omega^*|\omega)}\left|\frac{\partial(\omega^{a*})}{\partial(\omega^a)}\right|\right) \tag{2}$$

The terms in Eq.2 include:

- $p(\Omega^*|\mathbf{y})$ is the posterior distribution evaluated at the proposed state and $p(\Omega|\mathbf{y})$ is the posterior at the original state.
- $q(\omega^*|\omega)$ is the proposal density for the proposed state given the original state and $q(\omega|\omega^*)$ is the proposal density for the original state given the proposed state.
- $\left|\frac{\partial(\omega^{a*})}{\partial(\omega^a)}\right|$ is the Jacobian of the transition, with $\omega^a$ and $\omega^{a*}$ being parameter sets augmented with the random variables used to generate them.

$$\frac{q(\omega|\omega^*)}{q(\omega^*|\omega)} = \frac{q(n|n^*)}{q(n^*|n)}\frac{q(\mathbf{Z}|\mathbf{Z}^*)}{q(\mathbf{Z}^*|\mathbf{Z})}\frac{q(\mathbf{s}|\mathbf{s}^*,\mathbf{Z},\mathbf{Z}^*)}{q(\mathbf{s}^*|\mathbf{s},\mathbf{Z}^*,\mathbf{Z})} \tag{3}$$

The ratio of proposal densities can be expressed and factorized into a more meaningful form as provided in Eq.3. This equation reflects the manner in which the transition proposal is generated. First, either a birth or a death move is chosen from the

distribution $q(n^*|n)$. Then, either a new source location is proposed (for a birth move) or an existing location is randomly selected for deletion (for a death move) from the location proposal density $q(\mathbf{Z}^*|\mathbf{Z})$. Lastly, an updated set of source emission rates is sampled from the emission rate proposal density $q(\mathbf{s}^*|\mathbf{s}, \mathbf{Z}^*, \mathbf{Z})$.

In the case of a birth transition, the new source location is selected uniformly at random within the 3D box representing the site. In the case of a death transition, an existing source location is selected at random for deletion from the solution. For the emission rate transition, the full vector of emission rates is updated to ensure that the model predictions for the concentration data are unchanged after the transition. Following this, a small Gaussian perturbation is added to the emission rate corresponding to the new source location. Further details of this process are outlined in[4].

## 4 Controlled releases

This section provides additional plots characterizing the controlled releases analysis. Figure 5 shows the raw path averaged concentration data for the three releases on the 28th July. Figure 6 shows the 16-84% quantile range for the fixed grid joint inversion of the three releases from the 28th July and the release from the 4th August, and Figure 7 shows the fit to the data for the same inversion. Figure 8 shows the 16-84% quantile range for the reversible jump MCMC inversion of of the same releases, and Figure 9 shows the fit to the data from this RJMCMC inversion.

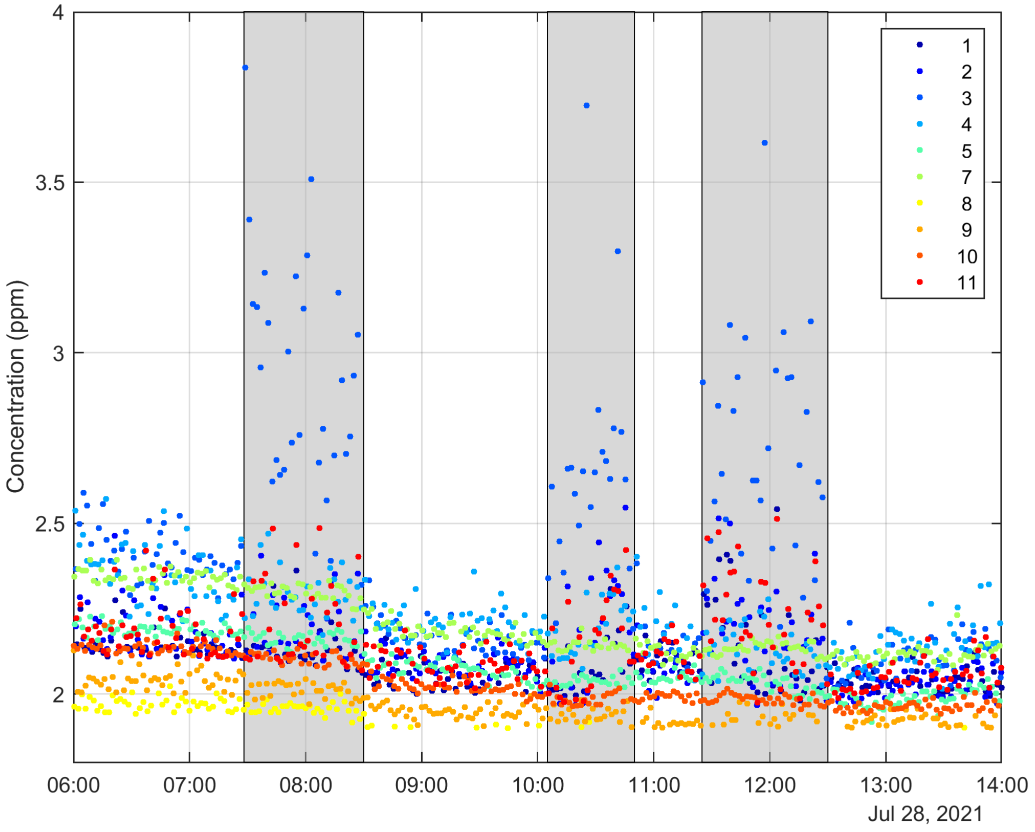


**Figure 5.** Path averaged Concentration data for the period from 06:00 to 14:00 UTC on 28th July 2021. Periods with a controlled release are highlighted in grey.

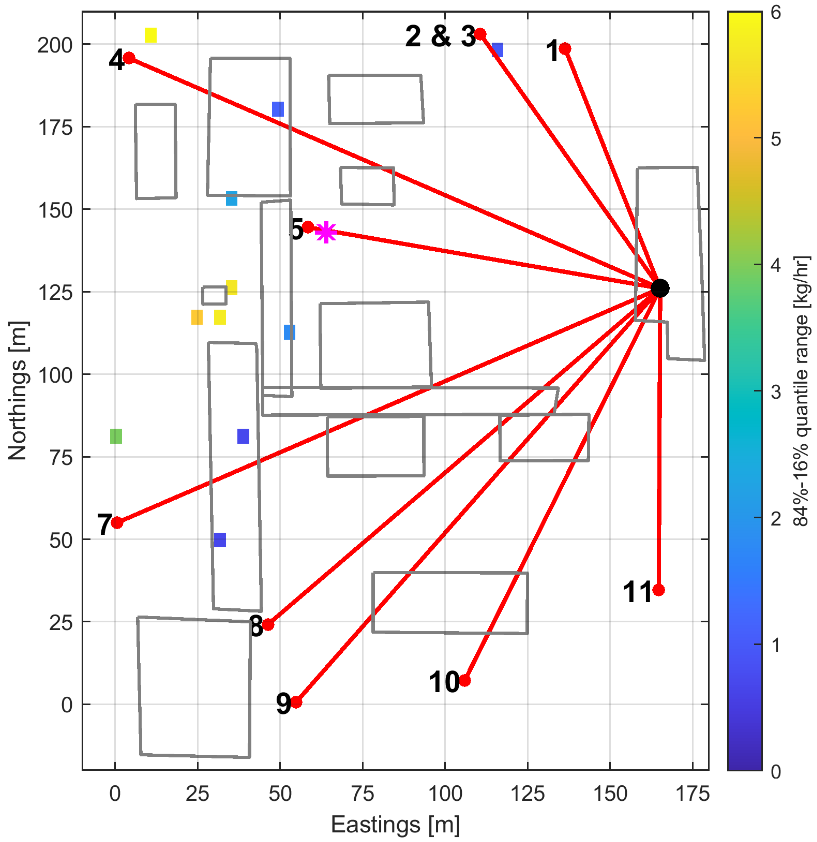


**Figure 6.** Case of the fixed grid inversion for the controlled releases on 28 July and 3 August 2021: this figure shows the 16-84% quantile range for each location.

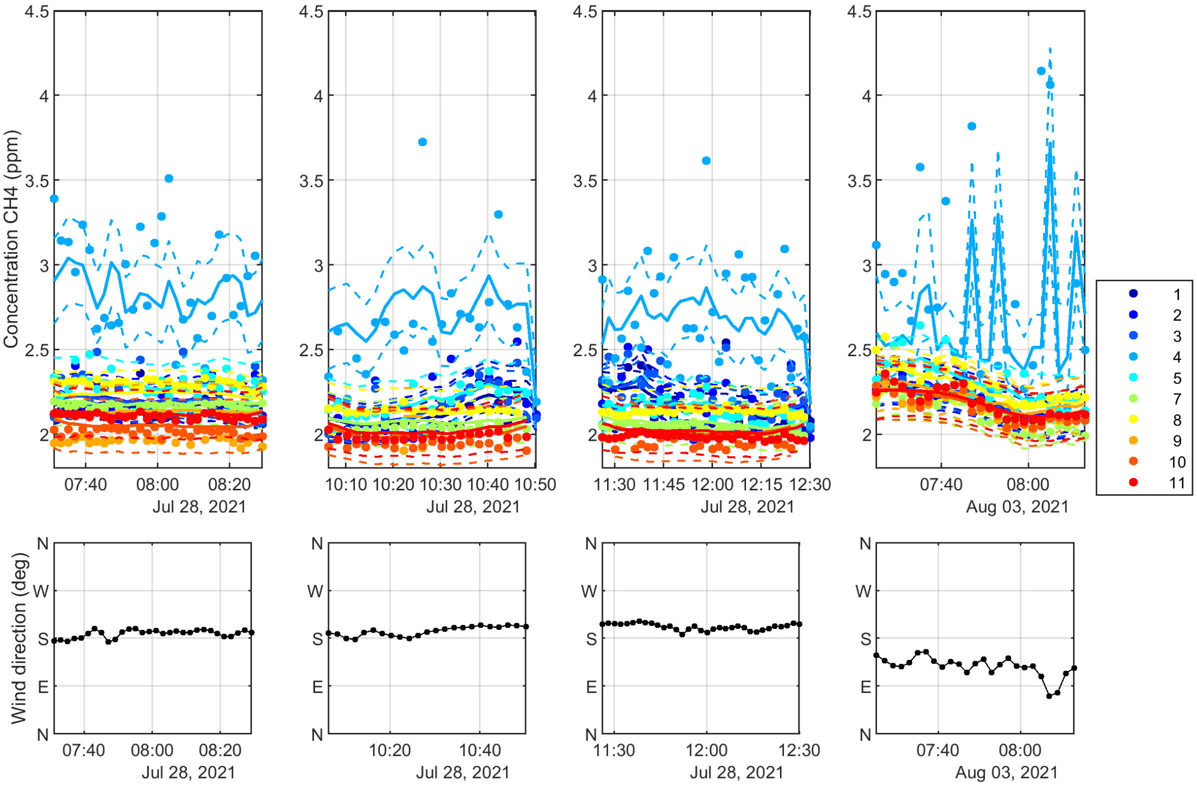


**Figure 7.** Data fit for the fixed grid inversion approach applied to the four controlled releases. The top panels show median, 2.5% and 97.5% concentration quantiles from the posterior MCMC iterations as lines, whilst the markers correspond to measured data. The lines and data points are coloured by beam. The bottom panels show the corresponding wind direction time-series for each of the four experiments.

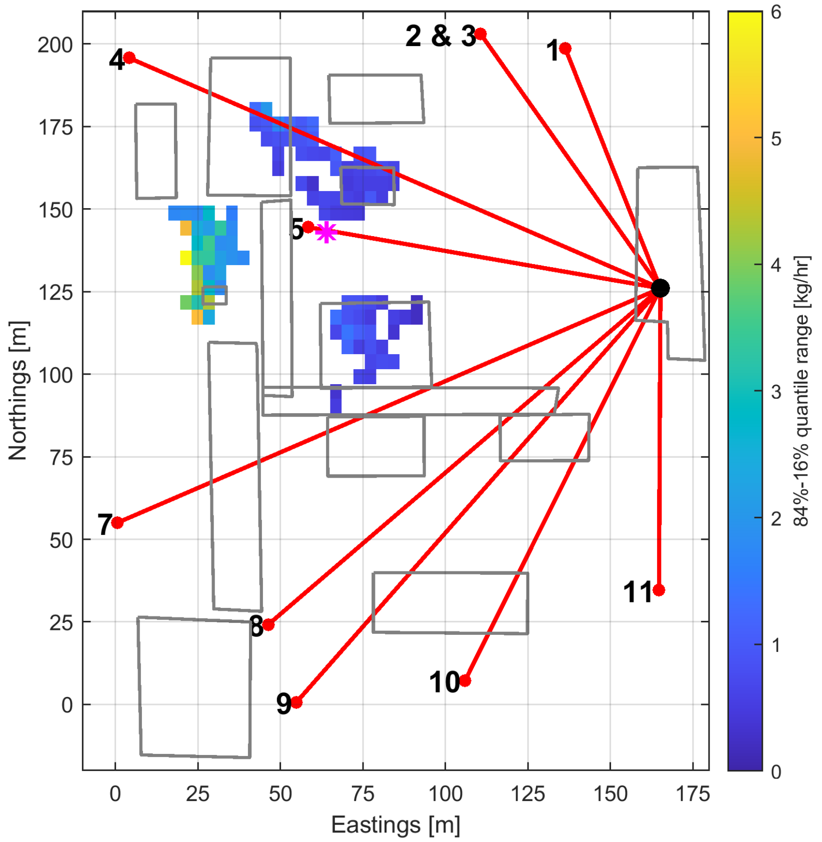


**Figure 8.** Map of the 16-84% quantile range corresponding to the RJMCMC posterior given in the main manuscript in the case of the controlled release.

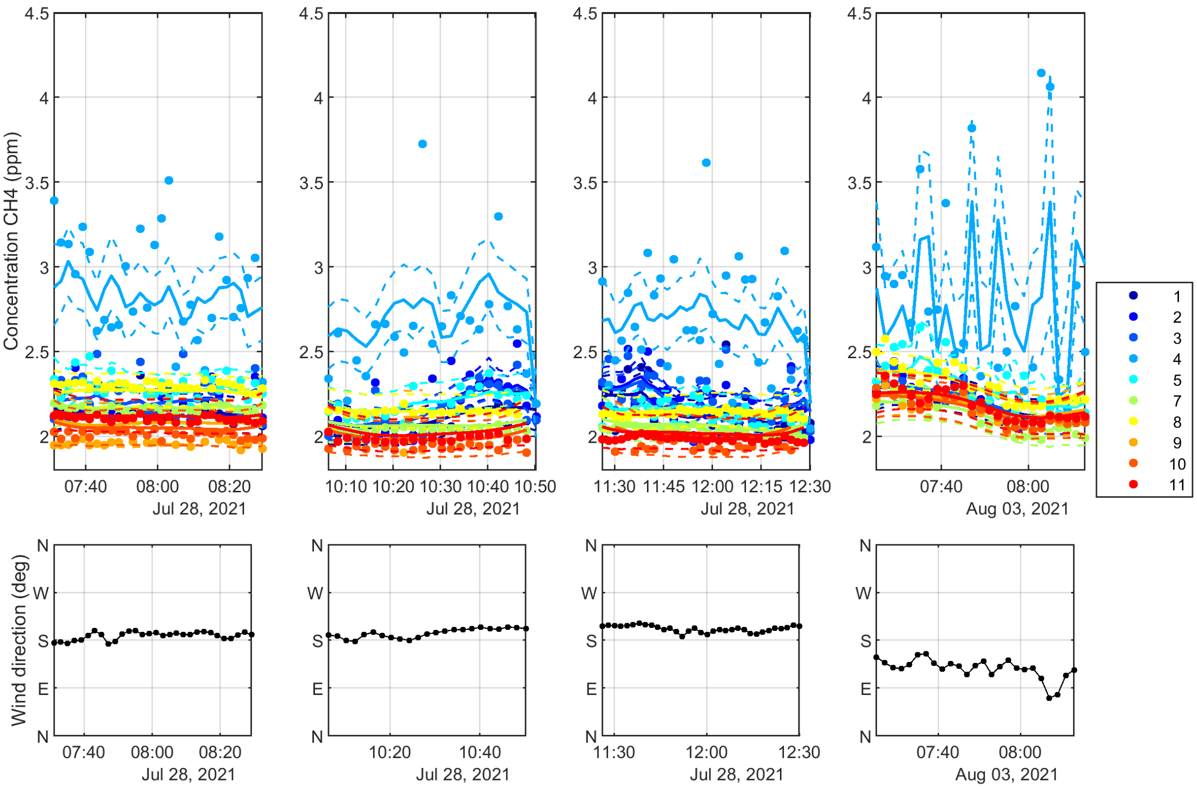


**Figure 9.** Data fit for the RJ inversion approach applied to the four controlled releases. The top panels show median, 2.5% and 97.5% concentration quantiles from the posterior MCMC iterations as lines, whilst the markers correspond to measured data. The lines and data points are coloured by beam. The bottom panels show the corresponding wind direction time-series for each of the four experiments.

## 5 System release

This section provides additional plots characterizing the system releases analysis. Figure 10 shows the 16-84% quantile range map for the reversible jump MCMC analysis of the system release data, and Figure 11 shows the fit to the data from this run.

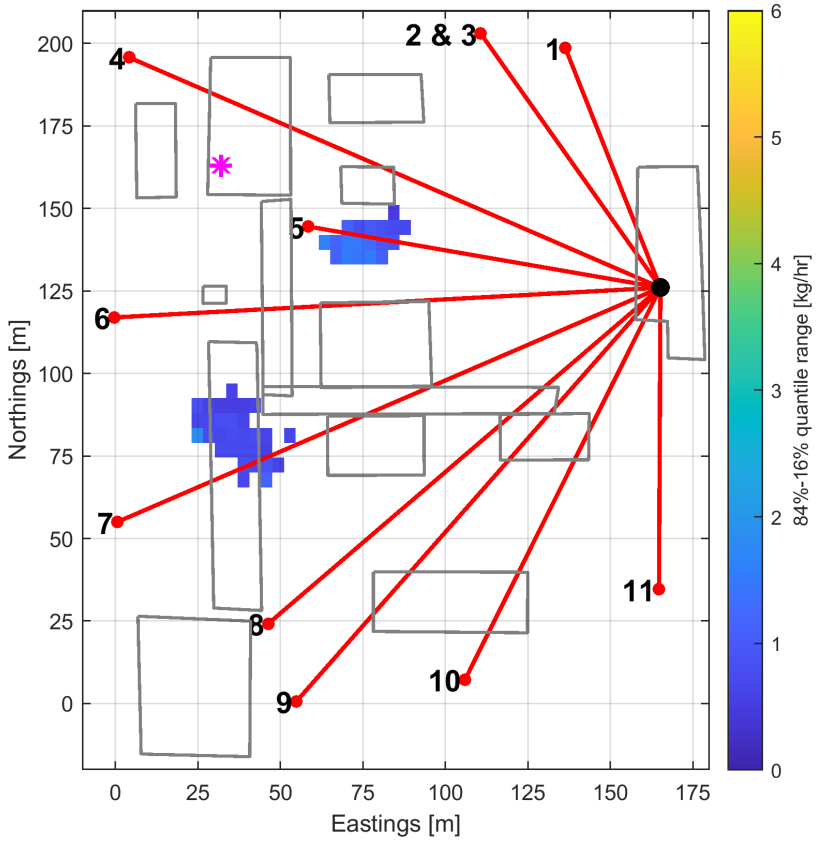


**Figure 10.** Map of the 16-84% quantile range corresponding to the RJMCMC posterior given in the main manuscript in the case of the system release from the 7th of July 2021.

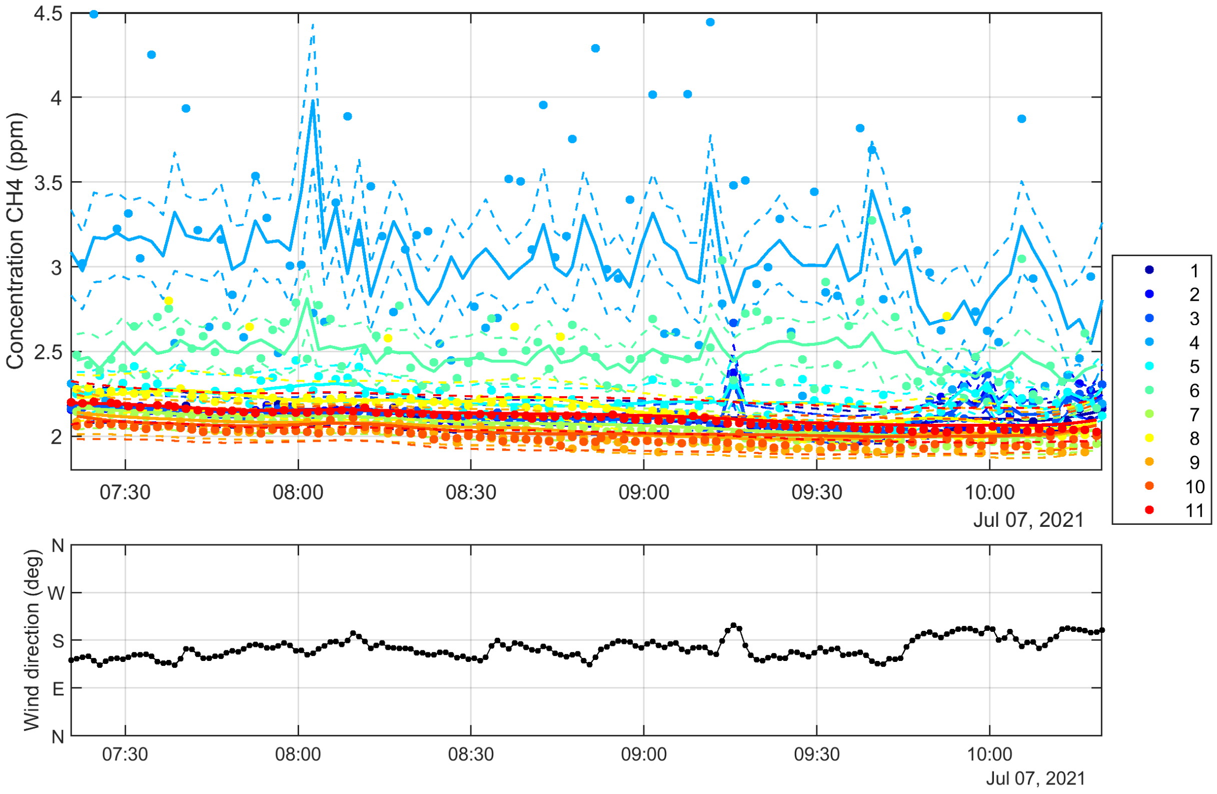


**Figure 11.** Data fit for the RJ inversion approach applied to the system release. The top panel shows the median, 2.5% and 97.5% concentration quantiles from the posterior MCMC iterations as lines, whilst the markers correspond to measured data. The lines and data points are coloured by the beam. The bottom panels show the corresponding wind direction time-series for this data segment.

## 6 Persistent emissions

This section provides additional plots characterizing the system releases analysis. Figure 12 shows the 16-84% quantile range maps for the fixed grid and reversible jump MCMC runs, and Figure 13b shows the data fit generated by each of these two runs.

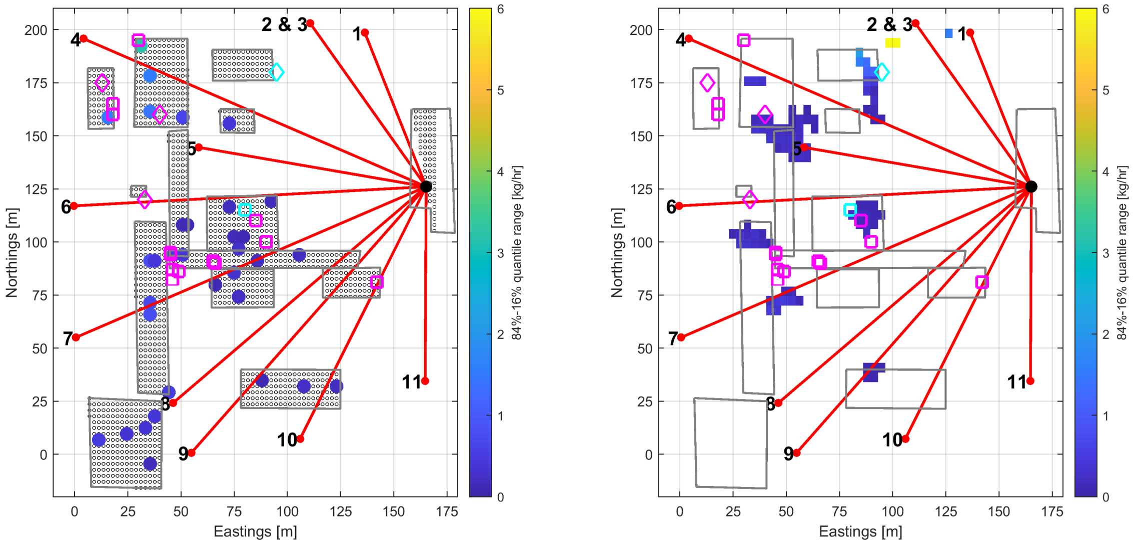


**(a)** Case of fixed grid MCMC.

**(b)** Case of reversibe jump MCMC.

**Figure 12.** Maps of the 16-84% quantile ranges corresponding to the posterior summaries presented in the main manuscript in the case of persistent emissions.

The outcomes from the fixed grid and reversible jump MCMC inversions were compared to that of the subsequent survey performed with an oil and gas industrial (OGI) camera. The comparison per equipment types is provided in Table 1.

| Facility equipment | Continuous (FG) | Continuous (RJ) | OGI camera survey |
|---|---|---|---|
| Compressor vent | no | yes | yes |
| Intercooler | yes | yes | no |
| Processing trains | yes | yes | yes |
| NW area with analyser house | yes | yes | yes |
| Piping and slug catcher | yes | yes | yes |
| Boiler | no | no | yes |
| Storage tank area | yes | no | not surveyed |
| SW area | no | no | no |

**Table 1.** Comparison of identified low intensity persistent emissions between the two types of MCMC inversion and the results from the subsequent OGI camera survey.

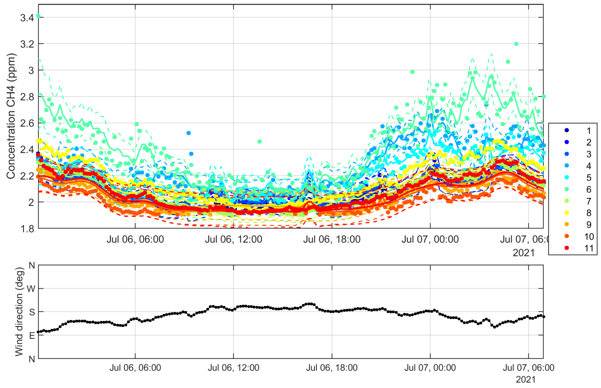


**(a)** Case of fixed grid MCMC.

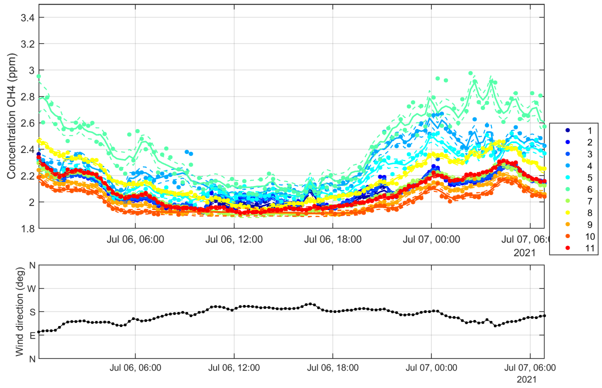


**(b)** Case of RJ MCMC.

**Figure 13.** Data fit from the MCMC runs for the persistent emission case. The top panel show median, 2.5% and 97.5% concentration quantiles from the posterior MCMC iterations as lines, whilst the dots corresponds to measured data. The lines and data points are coloured by the beam. The bottom panels show the corresponding wind direction time-series for the experiments.

## 7 June, August and September data set

The full data series are provided in the next pages.

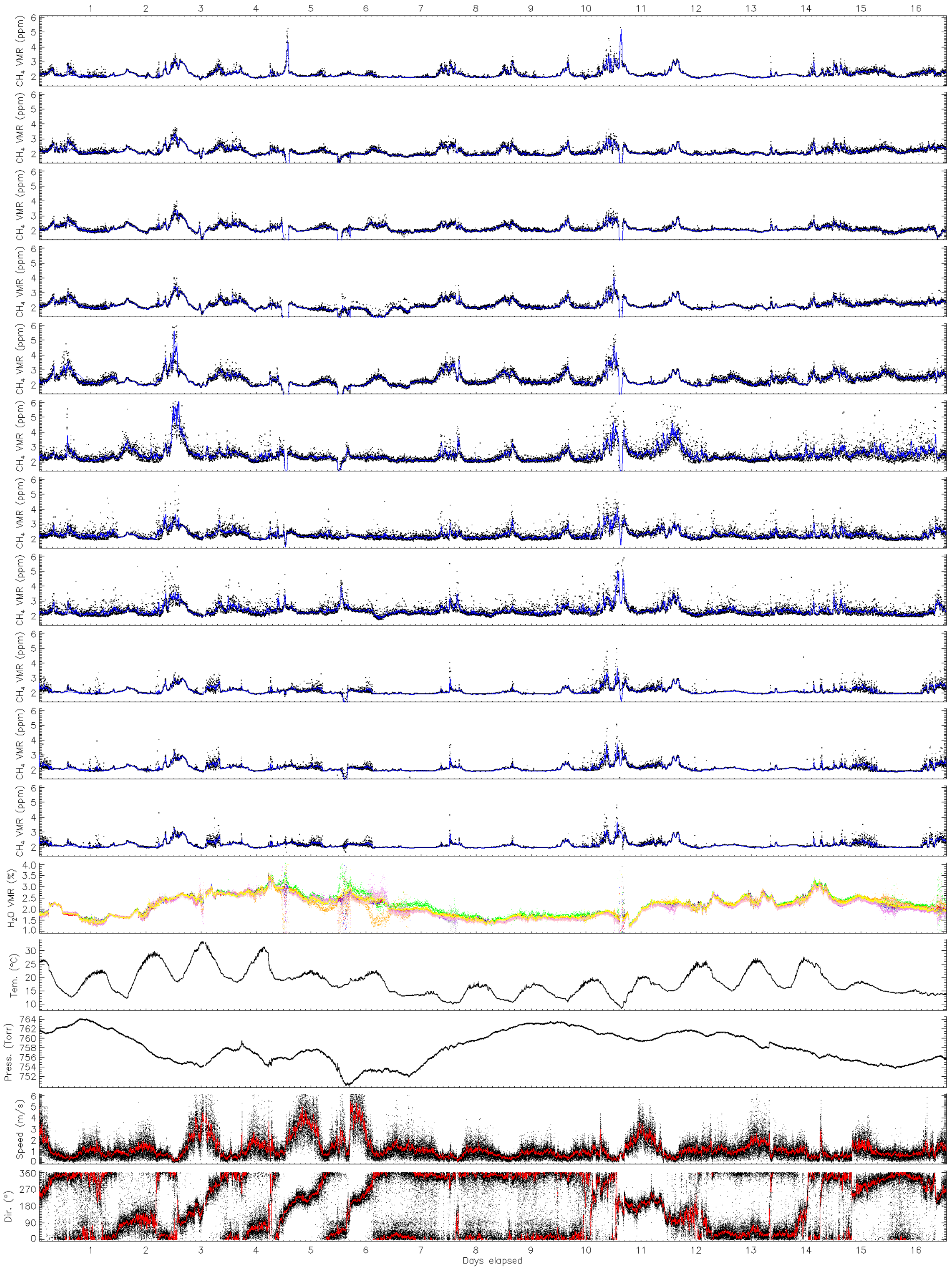


**Figure 14.** Subset of continuous measurements over the month of June 2021 of multi-path averaged concentrations of methane and associated meteorological data recorded by the sensor. The time ticks on the abscissa correspond to UTC noon for each day. The blue (red) line corresponds to a 10 (60) point smoothing.

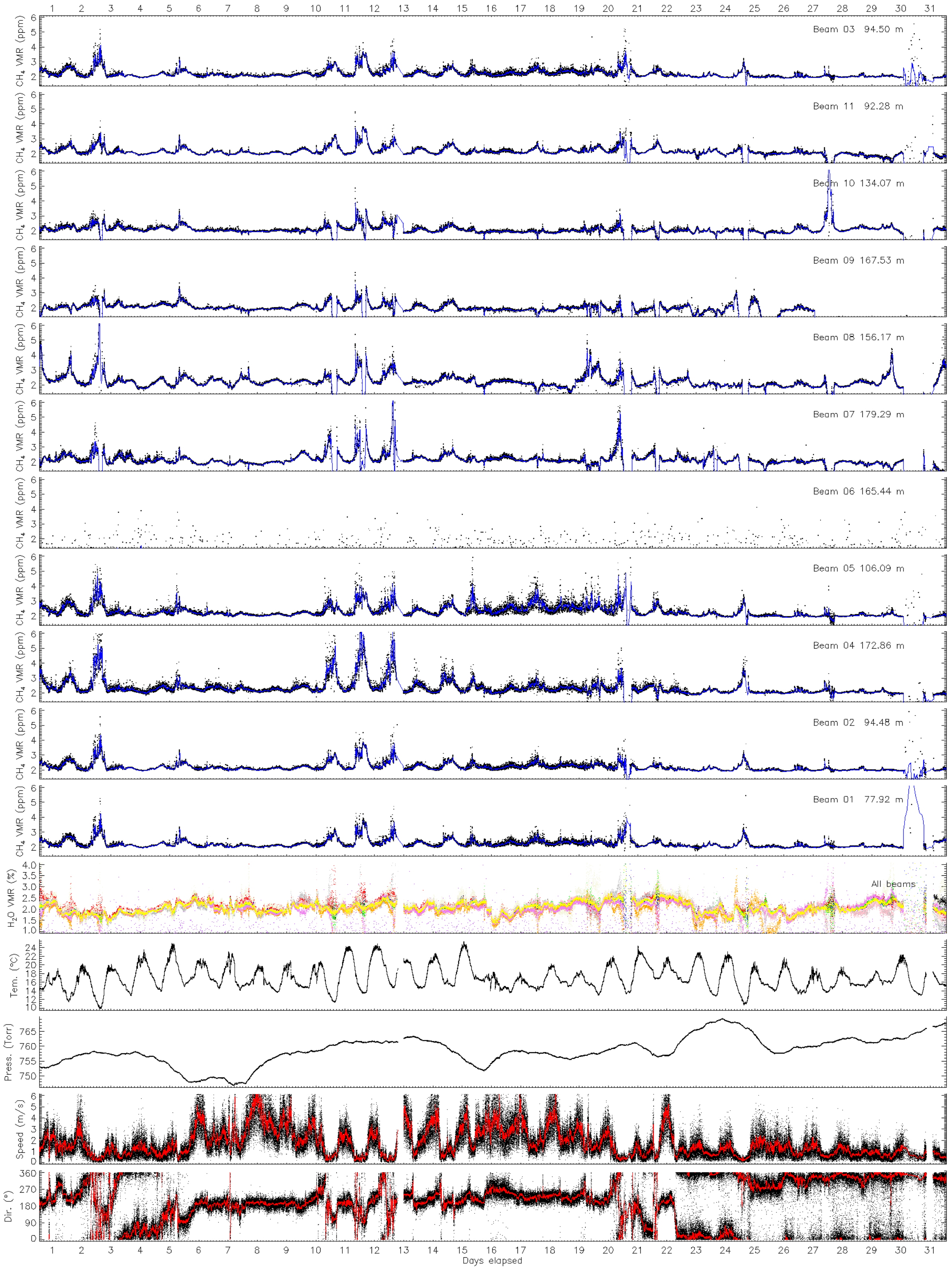


**Figure 15.** Subset of continuous measurements over the month of August 2021 of multi-path averaged concentrations of methane and associated meteorological data recorded by the sensor. The time ticks on the abscissa correspond to UTC noon for each day. The blue (red) line corresponds to a 10 (60) point smoothing.

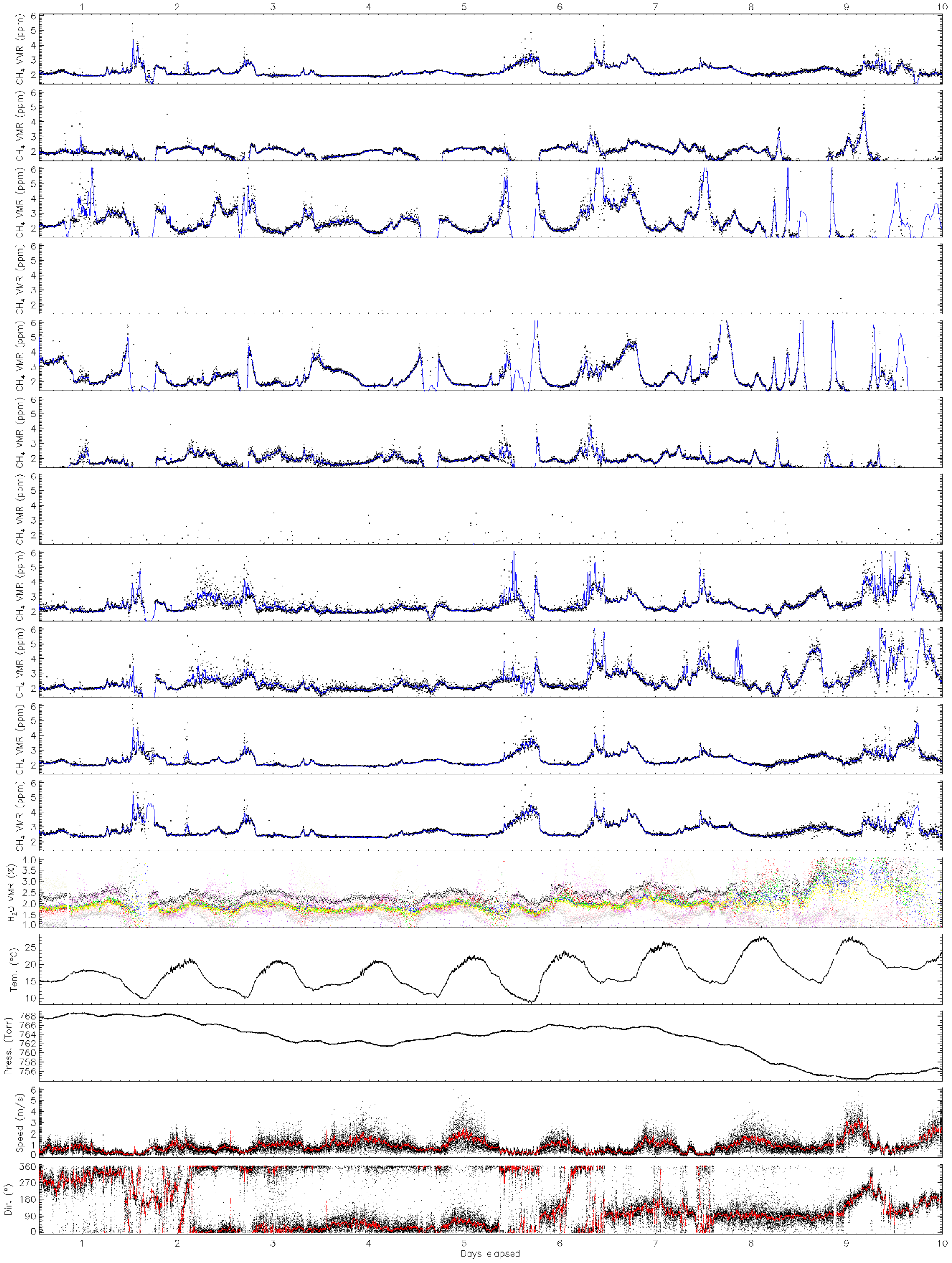


**Figure 16.** Subset of continuous measurements over the month of September 2021 of multi-path averaged concentrations of methane and associated meteorological data recorded by the sensor. The time ticks on the abscissa correspond to UTC noon for each day. The blue (red) line corresponds to a 10 (60) point smoothing.

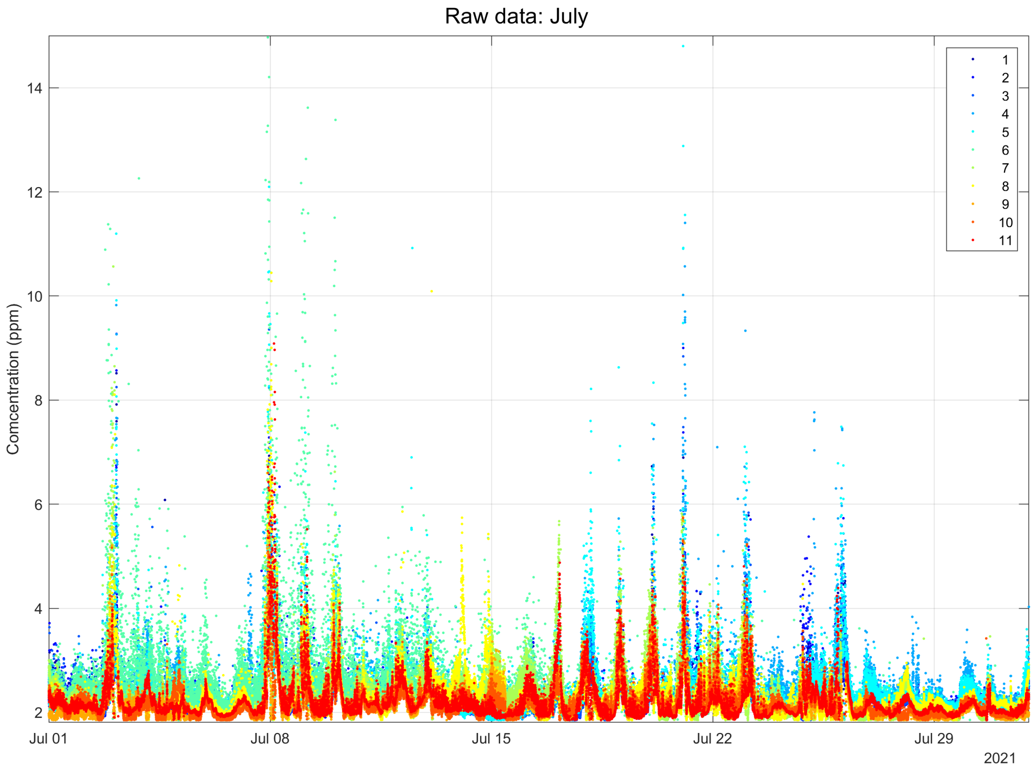


**Figure 17.** Subset of continuous measurements over the month of July 2021 of multi-path averaged concentrations of methane. The ordinate of the plot has been raised up to 15 ppm to show that measurements above 10 ppm occur during a nocturnal boundary layer trapping event, particularly for beam 6.

## References


1. Hirst, B. *et al.* Methane emissions: Remote mapping and source quantification using an open-path laser dispersion spectrometer. *Geophys. Res. Lett.* **47**, e2019GL086725, DOI: https://doi.org/10.1029/2019GL086725 (2020). E2019GL086725 2019GL086725, https://agupubs.onlinelibrary.wiley.com/doi/pdf/10.1029/2019GL086725.

2. Weidmann, D. *et al.* Locating and quantifying methane emissions by inverse analysis of path-integrated concentration data using a markov-chain monte carlo approach. *ACS Earth Space Chem.* **6**, 2190–2198, DOI: 10.1021/acsearthspacechem.2c00093 (2022). https://doi.org/10.1021/acsearthspacechem.2c00093.

3. Green, P. J. Reversible jump Markov chain Monte Carlo computation and Bayesian model determination. *Biometrika* **82**, 711–732, DOI: 10.1093/biomet/82.4.711 (1995). https://academic.oup.com/biomet/article-pdf/82/4/711/699533/82-4-711.pdf.

4. Zanini, E., Eastoe, E., Jones, M. J., Randell, D. & Jonathan, P. Flexible covariate representations for extremes. *Environmetrics* **31**, DOI: 10.1002/env.2624 (2020).
